# Landscape of somatic alterations in large-scale solid tumors from an Asian population

**DOI:** 10.1038/s41467-022-31780-9

**Published:** 2022-07-23

**Authors:** Liqun Wu, Herui Yao, Hui Chen, Aodi Wang, Kun Guo, Wenli Gou, Yanfei Yu, Xiang Li, Ming Yao, Shaohua Yuan, Fei Pang, Jinwei Hu, Lijuan Chen, Wenjin Liu, Jicheng Yao, Shuirong Zhang, Xiaowei Dong, Weifeng Wang, Jing Hu, Qi Ling, Songming Ding, Yan Wei, Qiang Li, Weichun Cao, Shuang Wang, Yang Di, Feiling Feng, Gang Zhao, Jian Zhang, Ling Huang, Jia Xu, Wangjun Yan, Zhongsheng Tong, Da Jiang, Tao Ji, Qiao Li, Ling Xu, Huiying He, Liang Shang, Jin Liu, Kefeng Wang, Duoguang Wu, Jingnan Shen, Ye Liu, Ting Zhang, Chaojie Liang, Yusheng Wang, Yanhong Shang, Jianji Guo, Guanbiao Liang, Shifeng Xu, Junfeng Liu, Kai Wang, Minghui Wang

**Affiliations:** 1 Department of Hepatobiliary Surgery, Affiliated Hospital of Qingdao University, Qingdao, China; 2 Guangdong Provincial Key Laboratory of Malignant Tumor Epigenetics and Gene Regulation, Department of Medical Oncology, Breast Tumor Center and Phase I Clinical Trial Center, Sun Yat-Sen Memorial Hospital, Sun Yat-Sen University, Guangzhou, China; 3 Shanghai OrigiMed Co., Ltd, Shanghai, China; 4 Liver Cancer Institute, Zhongshan Hospital, Fudan University, Shanghai, China; 5 Department of Obstetrics and Gynecology, First Affiliated Hospital of Xi’an Jiao Tong University, Xi’an, China; 6 Department of Medical Oncology, The First People’s Hospital of Yunnan Province, Kunming, China; 7 Department of Surgery, First Affiliated Hospital, School of Medicine, Zhejiang University, Hangzhou, China; 8 Department of Hepatobiliary and Pancreatic Surgery, Shu Lan (Hangzhou) Hospital, Hangzhou, China; 9 Department of Oncology, Guigang City People’s Hospital, The Eighth Affiliated Hospital of Guangxi Medical University, Guigang, China; 10 Department of Hepatobiliary Surgery, Affiliated Hospital of North Sichuan Medical University, Nanchong, China; 11 Department of Gynecologic Oncology, Hunan Cancer Hospital, The Affiliated Cancer Hospital of Xiangya School of Medicine, Central South University, Changsha, China; 12 Department of Pathology, School of Basic Medical Sciences, Southern Medical University, Guangzhou, China; 13 Department of Pancreatic Surgery, Huashan Hospital, Fudan University, Shanghai, China; 14 Department of Biliary Surgery, Shanghai Eastern Hepatobiliary Surgery Hospital, Shanghai, China; 15 Department of Pathology, Tianjin Medical University Cancer Institute and Hospital, Tianjin, China; 16 Department of General Surgery, Tongji Hospital, Tongji Medical College, Huazhong University of Science and Technology, Wuhan, China; 17 Department of Gastrointestinal Oncology, Huifuxi Branch of Guangdong Provincial People’s Hospital, Guangzhou, China; 18 Department of Emergency, The First Affiliated Hospital, Sun Yat-sen University, Guangzhou, China; 19 Department of Musculoskeletal Surgery, Shanghai Cancer Center, Fudan University, Shanghai, China; 20 Department of Breast Oncology, Tianjin Medical University Cancer Institute and Hospital, Tianjin, China; 21 Department of Oncology, Fourth Hospital of Hebei Medical University, Shijiazhuang, China; 22 Musculoskeletal Tumor Center, Beijing Key Laboratory for Musculoskeletal Tumors, People’s Hospital, Peking University, Beijing, China; 23 National Cancer Center/ Department of Medical Oncology, Cancer Hospital, Chinese Academy of Medical Sciences and Peking Union Medical College, Beijing, China; 24 Breast Disease Center, Peking University First Hospital, Beijing, China; 25 Department of Pathology, School of Basic Medical Sciences, Third Hospital, Peking University, Beijing, China; 26 Department of Gastrointestinal Surgery, Shandong Provincial Hospital Affiliated to Shandong University, Jinan, China; 27 Department of Gastroenterology, Shandong Provincial Hospital Affiliated to Shandong University, Jinan, China; 28 Department of Thoracic Surgery, Sun Yat-sen Memorial Hospital, Sun Yat-sen University, Guangzhou, China; 29 Department of Musculoskeletal Oncology, First Affiliated Hospital, Sun Yat-sen University, Guangzhou, China; 30 Department of Pathology, The Fifth Affiliated Hospital, Sun Yat-sen University, Zhuhai, China; 31 Department of Bone Marrow, The First Affiliated Hospital, College of Medicine, Zhejiang University, Hangzhou, China; 32 Department of General Surgery, The First Hospital of Shanxi Medical University, Taiyuan, China; 33 Department of Digestive, Shanxi Provincial Cancer Hospital, Taiyuan, China; 34 Department of Medical Oncology, The Affiliated Hospital, Hebei University, Baoding, China; 35 Department of Thoracic Surgery, First Affiliated Hospital of Guangxi Medical University, Nanning, China; 36 Department of Hepatobiliary Surgery, Shandong Provincial Hospital Affiliated to Shandong University, Jinan, China; 37 Department of Thoracic Surgery, Fourth Hospital of Hebei Medical University, Shijiazhuang, China; 38 Guangdong Provincial Key Laboratory of Malignant Tumor Epigenetics and Gene Regulation, Department of Thoracic Surgery, Sun Yat-Sen Memorial Hospital, Sun Yat-Sen University, 510120 Guangzhou, China

**Keywords:** Cancer genomics, Diagnostic markers, Medical research

## Abstract

Extending the benefits of tumor molecular profiling for all cancer patients requires a comprehensive analysis of tumor genomes across distinct patient populations worldwide. In this study, we perform deep next-generation DNA sequencing (NGS) from tumor tissues and matched blood specimens from over 10,000 patients in China by using a 450-gene comprehensive assay, developed and implemented under international clinical regulations. We perform a comprehensive comparison of somatically altered genes, the distribution of tumor mutational burden (TMB), gene fusion patterns, and the spectrum of various somatic alterations between Chinese and American patient populations. Here, we show 64% of cancers from Chinese patients in this study have clinically actionable genomic alterations, which may affect clinical decisions related to targeted therapy or immunotherapy. These findings describe the similarities and differences between tumors from Chinese and American patients, providing valuable information for personalized medicine.

## Abstract (web summary)

Understanding the mutation landscape of cancer may enable the development of more targeted therapies. Here, the authors sequence a panel of genes in a large Asian cohort and compare to American cohorts and find 64% of the Asian patients have actionable mutations.

## Introduction

Cancer morbidity and mortality remain a major challenge to public health in China, with over two million cancer deaths per year in China^1,2^. In recent years, precision oncology has enabled individual diagnosis, prognosis, and treatment based on increasingly accurate and high-resolution molecular stratification of cancers largely focused on genome targeted therapies^3,4^.

Next-generation sequencing (NGS) technology with the advantages of high throughput can identify all classes of genomic alterations on hundreds of genes including single nucleotide variant, insertion/deletion, copy number variation, and fusion/rearrangement at one time across multiple samples simultaneously. Considering the complexity of NGS technology and rigorous requirements of clinical practice, strict quality assurance and validation are necessary. For instance, the accreditation of the College of American Pathologists (CAP) or certification of Clinical Laboratory Improvement Amendments (CLIA) is a standard for effective verification. The previous study has shown that the NGS targeted CSYS assay for the clinical practices has been strictly validated^5^. Comparison with the F1CDx of Foundation Medicine which has been proved by the FDA also verifies the reliability of this CSYS assay^6^. The recognition of these panel NGS technologies provides the possibility for large-scale genomic characterization of cancer patients.

Patient ethnicity can also be a factor in cancer diagnostics and treatment since differences in cancer gene alterations exist between populations of various ethnicities^7–9^. A number of large-scale NGS pan-cancer studies on the Western population have been reported and displayed on cBioPortal^10^. It is blank for the Asian population, although many studies focusing on particular tumor types have been performed. In this work, we collected both tissue and blood samples from over 10,000 solid tumor patients and identified genomic alterations by using the previously validated clinical NGS panel and elaborated on the different genomic characteristics of Eastern and Western tumor patients comprehensively. This is the large-scale molecular profiling study of Asian solid tumor patients by deep sequencing of hundreds of cancer genes from both tissue and blood samples in a validated lab, and clinical significance interpretation of comprehensive genomic alteration detection and precision medicine.

## Results and discussion

### Description of the cohort

**Fig. 1:**
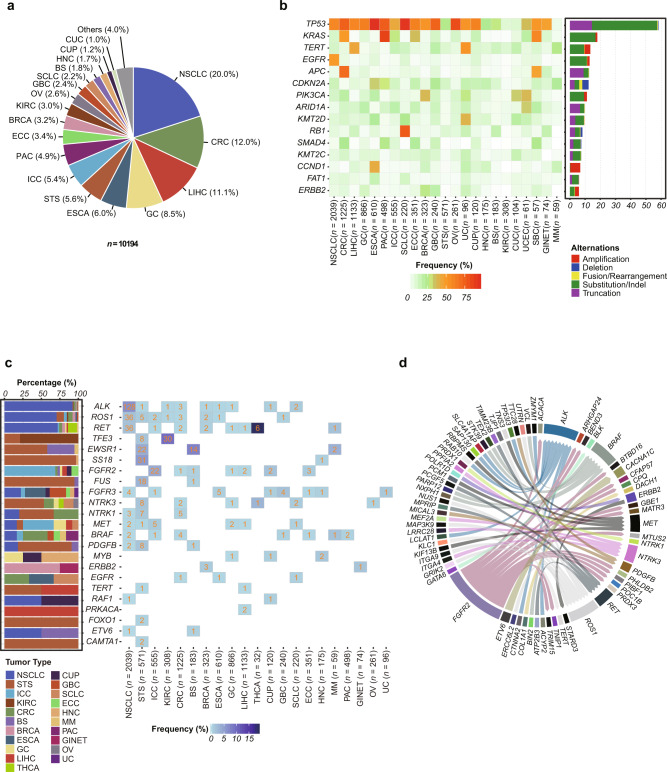
Overview of the mutational landscape of solid tumors from Chinese patients. **a** Distribution of tumor types among cases successfully sequenced from 10,194 patients. **b** Recurrent somatic alterations across common tumor types. The top 15 Tier 1 Cancer Gene Census oncogenes and tumor suppressor genes with a cohort-level variation frequency of ≥6% were shown, in descending order. Bars represent the percent of cases within each tumor type having at least 1 of 5 different classes of genomic alterations. **c** Genes recurrently rearranged to form putative gene fusions were displayed across principal tumor types. The tumor type-specific distribution of these genes was presented on the left side (various colors represent different tumor types). The number of corresponding gene fusions in each tumor type was shown in the right boxes, and the frequency was shown in gradient blue. **d** Gene fusions across multiple tumor types. A total of 57 driver-partner relationships were detected spanning 71 genes. The thickness of the line between two genes implied the relative count.

To explore the genomic landscape of Chinese patients with solid tumors as encountered in clinical practice, we collected tumor specimens and matched peripheral blood specimens from 11,553 individuals encompassing 25 principal tumor types and more than 100 tumor subtypes. After excluding samples (*n* = 1359) with insufficient tumor content or DNA yield or subsequent technical failure (Supplementary Fig. 1a), we successfully sequenced 10,194 (88%) tumor samples. In order to reduce statistical bias, the cancer types with <50 cases were excluded from the analysis. Summaries of the clinical characteristics of the patients’ specimens and the median sequencing target coverage of samples are presented in Supplementary Data 1 and 2 and Supplementary Figs. 2 and 3. A total of 31 ethnicities were presented in our cohort with Han being the most frequent (92%, 9382/10,194). The majority of patients in this study were from eastern and southern provinces in China (“East China” and “South China” in Wikipedia) (41 and 29%, respectively). In terms of tumor stage, 55% (5652/10,194) of patients had advanced-stage cancers (stage III/IV), while 35% (3579/10,194) had early-stage cancers (pre-cancers or stage I/II). In our entire cohort, majorities (76%) of patients were treatment-naive, and patients with previous treatments accounted for 16%. The remaining 8% of patients do not have confirmed or available treatment history information (Supplementary Fig. 1b). The major tumor types were non-small cell lung cancer (NSCLC; 20%), colorectal carcinoma (CRC; 12%), liver hepatocellular carcinoma (LIHC; 11%), gastric cancer (GC; 8%), esophageal carcinoma (ESCA; 6%), soft tissue sarcoma (STS; 6%), intrahepatic cholangiocarcinoma (ICC; 5%), pancreatic cancer (PAC; 5%), extrahepatic cholangiocarcinoma (ECC; 3%), and breast carcinoma (BRCA; 3%) (Fig. 1a). In general, the distribution of these predominant tumor types such as liver cancer (LIHC, ICC, and ECC) and lung cancer (NSCLC and SCLC) represented the distribution of tumors and mortality encountered in clinical practice in China^1^.

### Mutation landscape and gene fusions

Based on an NGS-based assay with a validated 450-gene panel^5^, we detected 80,703 single nucleotide variants (SNVs) and insertions and deletions (InDels), 19,192 truncations, 17,779 gene amplifications, 1688 gene homozygous deletions, and 3111 gene fusions/rearrangements in the 10,194 cases. We only focused on somatic alterations within tumors in this study, without germline genetic data. Analysis of significantly mutated cancer-related genes in solid tumors found the most frequently altered genes to be *TP53* (58% of cases), *KRAS* (18%), *TERT* (14%), *EGFR* (13%), *APC* (13%), *CDKN2A* (12%), and *PIK3CA* (11%). The most common mutations were *KRAS*^G12^, *EGFR*^L858^, and *TP53*^R273^ (Fig. 1b and Supplementary Data 3). The top three hotspots of *TP53* mutations were R273, R175, and R248. R175H/L was the most common mutation in CRC (8%) and PAC (6%), and R249S was detected in HCC (10%) and ICC (3%) respectively, which was rarely observed in other tumor types (Supplementary Fig. 4 and Supplementary Data 4). Of note, *EGFR* and *KRAS* represented obviously pairwise co-occurring alterations with SNVs/InDels and copy number variation (CNV) (Supplementary Fig. 5). Subsequent analysis of CNV showed high frequencies of *CDKN2A/B* deletion, *SMAD4* deletion, *ERBB2* amplification, *EGFR* amplification, and *MYC* amplification in metastatic samples, and chromosome 11q13.3 (*CCND1*/*FGF3*/*FGF4*/*FGF19*) amplification in primary samples at the pan-cancer level. Meanwhile, we found that *ERBB2* amplification and chromosome 11q13 amplification were respectively enriched in breast cancer (BRCA) (24 vs. 2%; FDR = 7.645E−105) and ESCA (43 vs. 4%; FDR = 3.553E−301), compared to other tumor types (Supplementary Fig. 6 and Supplementary Data 5 and 6). In addition, we sought to investigate the features of gene fusions in solid tumors and identified a total of 513 fusion events, including 31 driver genes in our cohort. As shown in Fig. 1c, fusion events in genes such as *ALK* (*n* = 139), *ROS1* (*n* = 51), *RET* (*n* = 50), *FGFR2*/*3* (*n* = 50), *NTRK1*/*3* (*n* = 30), and *BRAF* (*n* = 12) occurred widely across tumor types, while others such as *EWSR1* and *TFE3* were enriched in certain tumor types (sarcomas [soft tissue sarcoma or STS, and bone sarcoma] and KIRC, respectively). *PRKACA* fusions were only detected in a specific tumor type (LIHC, subsequent diagnosis as fibrolamellar hepatocellular carcinoma [FL-HCC]). Moreover, rarely reported fusion partners for driver genes were identified, including multiple fusions in kinase genes such as *GRIK2-ROS1*, *PARP12-BRAF*, *KIF13B-MET*, and *LRRC28-NTRK3*, and fused exons in *KIF5B-ALK* and *EML4-ALK* compared to the Quiver database (http://quiver.archerdx.com/) (Fig. 1d, Supplementary Data 7, and Supplementary Figs. 7 and 8).

### Clinical features and related altered genes

**Fig. 2:**
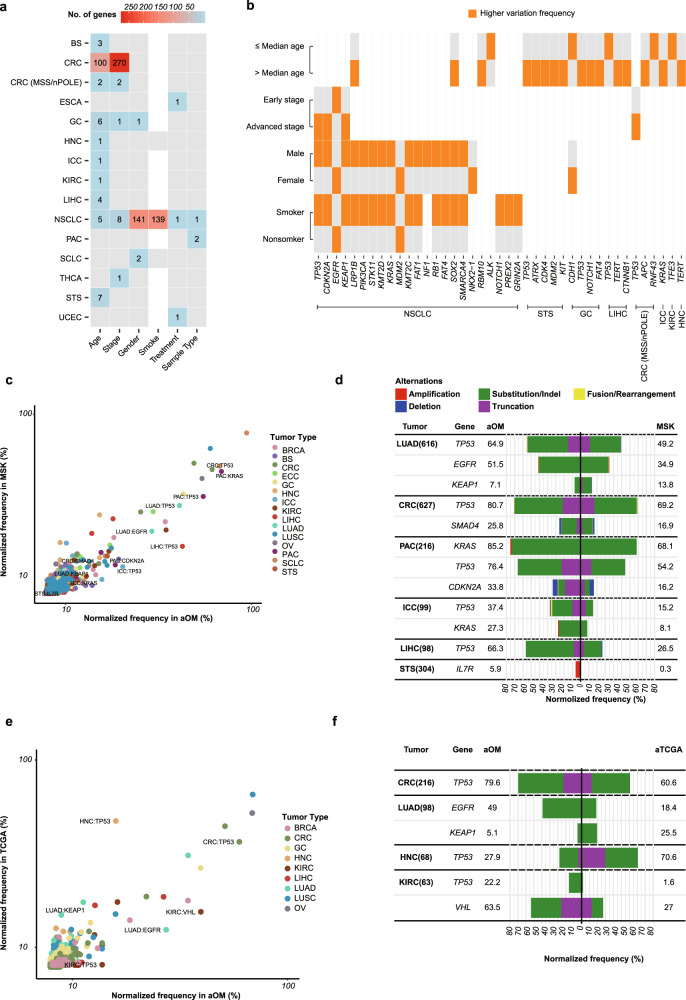
Analysis of somatic altered genes. **a** Numbers of correlated altered genes with six clinical features across tumor types. Only genes with significant differences (FDR < 0.05) between two groups of clinical features were calculated. The “age” feature included the younger patient group and the older patient group, separated by the median initial diagnosis age of patients of each tumor type. The “stage” feature included early-stage cancer group and advanced-stage cancer group. The “smoke” feature, including the smoker group (current smokers and former smokers) and nonsmoker group (never-smokers), was analyzed in lung cancers (NSCLC and SCLC) and head and neck cancers (HNC). The “treatment” feature included treatment-naive group and the pretreated group. The “sample type” feature included the primary sample group and metastatic/recurrent sample group. **b** Correlation between Tier 1 Cancer Gene Census genes and clinical features. Genes with significant differences (FDR < 0.05, number of each group >60, and sum of variation frequencies >10%) between two feature groups were shown. The group with a higher variation frequency in each clinical feature was labeled in orange. **c** Frequency of altered gene in 15 comparable tumor types between the aOM cohort and MSK cohort. **d** Comparison of significantly different altered genes (FDR < 0.05) between the aOM cohort (left) and MSK cohort (right). Altered genes whose sum of frequencies in the two cohorts were displayed. The alteration frequencies (%) of specific genes were shown in the “aOM” and “MSK” columns. **e** Frequency of altered gene in 9 comparable tumor types between the aOM cohort and aTCGA cohort. **f** Comparison of significantly different altered genes (FDR < 0.05) between the aOM cohort (left) and MSK cohort (right). Altered genes whose sum of frequencies in the two cohorts were displayed.

To reveal further somatic alterations associated with clinical characteristics in Chinese cancer patients, we implemented an integrative analysis across the tumor-type distribution of genomic profile and six clinical features including age, gender, tumor stage, smoking history (only in NSCLC, SCLC, and HNC), treatment and sample type (primary vs. metastatic/recurrent) (Supplementary Data 8 and Fig. 2a, b). In general, clinical feature-associated genomic differences were observed distributed in CRC and NSCLC. In CRC, the number of differentially mutated genes were respectively 270 and 100 in younger and early-stage patients as compared to older and advanced-stage patients, which could be consistent with the significantly high ratio of hypermutated subtypes, such as microsatellite instability-high (MSI-H) and *POLE*-associated CRC (with microsatellite stability [MSS], high mutation burden and an inactive *POLE* mutation) in younger and early-stage CRC (Supplementary Fig. 9, *FDR* < 0.05). In NSCLC, the frequency of mutated genes was markedly affected by gender and smoking history. Of note, gender and smoking history were not independent factors in our cohort, because the majority of nonsmokers were female. It was found that female nonsmokers with early-stage NSCLC harbored more mutations in *EGFR*, while male smokers with advanced-stage NSCLC were characterized by more mutations in *TP53*, *CDKN2A*, *PIK3CA*, and *KRAS*, consistent with recent reports^11^. Moreover, younger female gastric cancer patients had more *CDH1* mutations. In contrast, older gastric cancer patients tended to have more mutations in *TP53*, *NOTCH1*, and *FAT4*. In addition, younger LIHC, KIRC, and bone sarcoma patients harbored, respectively, *TP53*, *TFE3*, and *VEGFA* mutations, while older LIHC, HNC, and STS patients had, respectively, *CTNNB1*, *TERT*, and *TP53* mutations (Fig. 2b, FDR < 0.05).

### Comparison of the frequency of somatically gene mutations

To assess the characteristics of cancer genomes from Chinese patients in a global context, we made a comparison of genomic alterations with the largest published cancer genomic study of the Memorial Sloan Kettering Cancer Center (MSK) IMPACT study^12^, including 10,366 cases, mostly advanced cancer specimens. 266 genes in common between the two platforms were compared in 15 comparable advanced-stage tumor types between the advanced OrigiMed (OM) cohort (aOM, *n* = 2820) and MSK cohort (*n* = 2820). To limit the bias of comparisons, we subdivided NSCLC of the two cohorts into lung adenocarcinoma (LUAD) and lung squamous cell carcinoma (LUSC) and we used PSM (Propensity Score Matching) to balance available clinic confounder factors in different cohort such as primary/metastasis/recurrent tumor specimens, sampling method, gender, and smoke. Overall, only 12 tumor type: gene pairs presented significant differences in the frequency of gene variants between the aOM cohort and the MSK cohort (FDR < 0.05) (Fig. 2c, d and Supplementary Data 9), suggesting frequencies of the most common mutated genes and the tumor-type distribution in aOM cohort were highly consistent within the MSK cohort, such as CRC: *APC* (71.9 vs. 72.7%, FDR = 1) and SCLC: *RB1* (84.2 vs. 71.1%, FDR = 1). The significant differences between the two cohorts were mainly found in lung adenocarcinoma and hepatobiliary tumors, such as LUAD: *EGFR*, ICC: *KRAS*. Moreover, several gene fusions and CNVs also showed differences between the aOM cohort and the MSK cohort.

To further confirm the similarities and differences between the OM and MSK studies observed in advanced cancers, we also compared the aOM data with genomic data of advanced-stage cases from The Cancer Genome Atlas studies (aTCGA). Because of heterogeneous methodologies (including detecting platform, algorithm, and report criteria of variants), SNVs, InDels and truncations mutations were considered in the comparison. In 9 comparable tumor types and 266 genes, we identified a total of 6 tumor types: gene pairs with significant differences between the aOM cohort (*n* = 1008) and the aTCGA cohort (*n* = 1008) (FDR < 0.05) (Fig. 2e, f and Supplementary Data 10), of which 3 different tumor type: gene pairs presented consistently changing trends with those in the comparison between the aOM cohort and the MSK cohort, including higher frequencies of CRC: *TP53* and LUAD: *EGFR* and lower frequencies of LUAD: *KEAP1* in the aOM cohort, compared with other two cohorts. Altogether, these multiple comparisons revealed at the greatest extent the similarity and distinctive of genomic alteration across these cohorts.

### Immunotherapy-related biomarkers

**Fig. 3:**
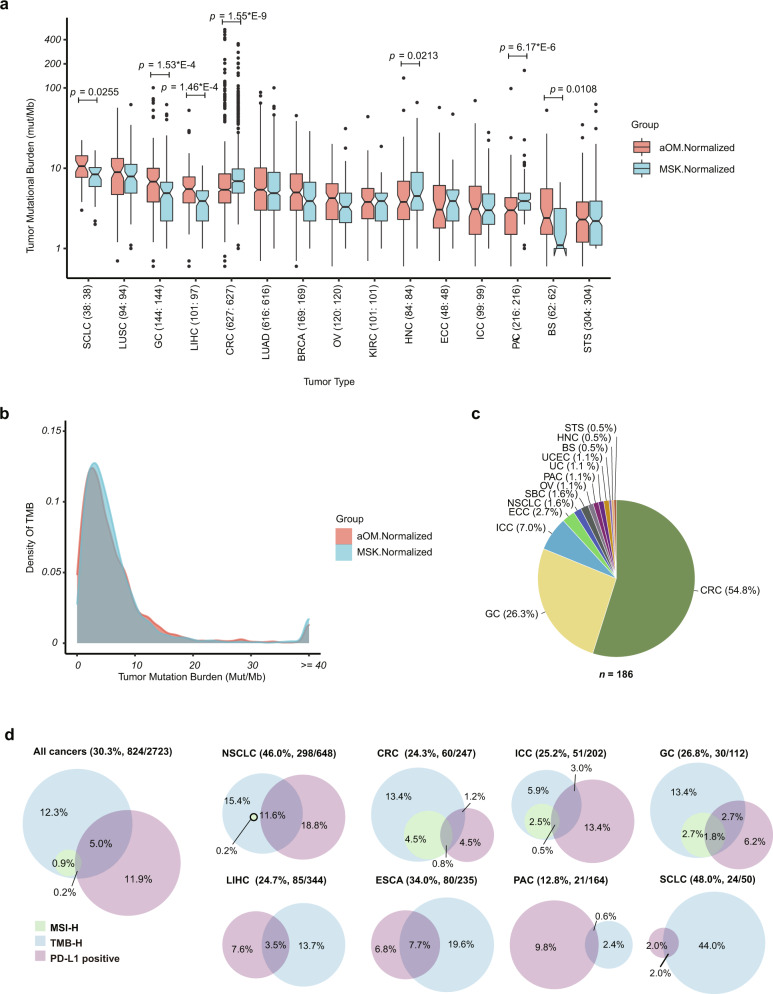
Correlation of tumor mutational burden (TMB), microsatellite instability high (MSI-H), and PD-L1 expression in OM cohort. **a** The tumor-type-specific distribution of TMB (excluding samples with TMB of 0) between the aOM cohort (light red) and the MSK cohort (light blue). Tumor types were sorted from left to right based on median TMB values (y-axis). The total number of samples was shown for each tumor type. *P* values were labeled on the top of corresponding tumor types in which TMB was significantly different between cohorts and calculated using a two-sided Wilcoxon rank-sum test. The boxplot elements indicate the maxima, 75th percentile, median, 25th percentile, and minima. Notches are used to compare groups; if the notches of two boxes do not overlap, this suggests that the medians are significantly different. **b** Distribution of TMB density between the aOM cohort (light red) and the MSK cohort (light blue). **c** The tumor-type-specific distribution for 186 samples with MSI-H. **d** The analysis for the cohort-level or tumor type-specific correlation of TMB, MSI, and PD-L1 expression in 2,723 samples with available information on MSI, TMB, and PD-L1. The Venn diagram showed the proportion of TMB-H (light blue), PD-L1 positive (light purple), and MSI-H (light green). Total proportions, numbers of samples with at least MSI-H, TMB-H, or PD-L1 positive, and total numbers of samples were shown in parentheses.

In addition to targeted therapy, the recent clinical success of immune checkpoint blockade^13–16^ makes the comparison of immunotherapy-related mutations and signatures across cancers from patients in different countries another important question. Hence, we analyzed the distribution of tumor mutational burden (TMB) within tumor types. Even though an algorithm to evaluate TMB in routine clinical practice has not yet reached a consensus^17^, an individual TMB has been shown to predict patient outcomes after immunotherapy^13–16^. Here, we identified TMB high (TMB-H) and TMB low (TMB-L) according to the TMB-high status definition from the KEYNOTE-158 study (the value ≥10 Muts/Mb or not)^16^. As shown in Fig. 3a and Supplementary Data 11, median TMB values in nearly half tumor types in the aOM cohort were different compared with the MSK cohort (Supplementary Fig. 10). Overall, the whole pattern of TMB distribution in the aOM cohort was similar to that in the MSK cohort, characterized by a “tail” that includes 119 samples with TMB ≥ 40 (Fig. 3b). We further analyzed the distribution of 186 samples harboring MSI-H in our cohort and found that the overall proportion of patients with MSI-H was 2% and was mainly in CRC (55%, 102/186) (Fig. 3c). Previous studies have suggested that TMB and PD-L1 expression are two independent biomarkers, and there is no significant correlation between PD-L1 expression and TMB in most cancer subtypes^18,19^. However, because MSI-H and TMB-H have recently been recognized as biomarkers for response to immune checkpoint blockades (anti-PD-1/PD-L1)^13,16^, we evaluated the combined association of TMB and MSI with PD-L1 expression evaluated by immunohistochemically (IHC) staining in 2723 tumors of OM cohort. The overall proportion of samples with at least one MSI-H, TMB-H, or PD-L1 positive was 30.3% (824/2723). SCLC harbored the highest proportion of samples with at least one MSH-H, TMB-H, or PD-L1 positive (48%; 24/50), followed by NSCLC (46%; 298/648), and ESCA (34%; 80/235) (Fig. 3d and Supplementary Fig. 11), suggesting the possibility of a high proportion of Chinese patients with lung cancer benefitting from immunotherapy.

In addition, recent evidence has suggested somatic amplification in the gene for programmed cell death ligand 1 (PD-L1/*CD274*) as a response biomarker to immunotherapy in solid tumors, even in the absence of MSI-H, PD-L1 overexpression or TMB-H^20^. Herein, we identified a total of 85 (1%) tumors with *CD274* amplification (copy number ≥6) in the OM cohort, a proportion consistent with a previous study^21^ (Supplementary Fig. 12a). Furthermore, in 30 evaluable samples with *CD274* amplification tested for PD-L1 expression, the PD-L1 positive rate was 70% (Supplementary Fig. 12b). Subsequently, we also examined the mutational landscape of the 85 samples with *CD274* amplification and found the co-occurrence of *CD274* amplification with adjacent *PDCD1LG2* and *JAK2* amplification (89% and 82% respectively), which are nearby genes in chromosome 9p24.3-9p22.2, associated with advanced stage and poorer outcome^21^. A high frequency of *TP53* mutations (78%) was also observed in these tumors (Supplementary Fig. 12c).

### Clinically actionable alterations

**Fig. 4:**
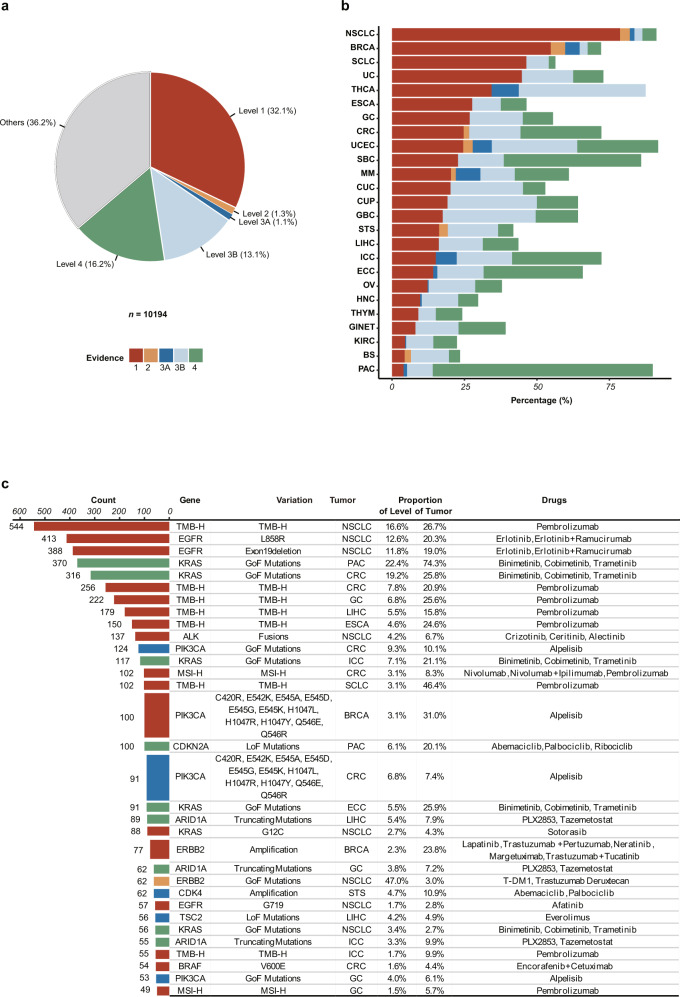
Clinical actionability of somatic alterations in the OM cohort. **a** Variants were assigned to different levels of clinical actionability according to OncoKB. The distribution of the highest level of actionable variants across all patients was shown in the pie chart. The colors representing each level were used throughout the other panels in this figure. **b** Distribution of highest level of actionable variants across tumor types. **c** Details of the 30 most common actionable variants, proportions of levels in corresponding tumor types, and their potential sensitive drugs. The numbers of patients in each level were shown in the bar graph. The right table showed genes, variants, and the tumor type for each level of clinical actionability, as well as the proportion of patients in the corresponding tumor type and level. Potential sensitive drugs suggested by biomarkers were also shown in the table.

To assess the potential clinical impact of the somatic alterations found in our cohort, we used the MSK criteria^12,22^ to systematically evaluate actionable variants identified in solid tumors from Chinese patients in our cohort, using the OncoKB (http://oncokb.org/, v3.6) knowledge base. Patients who harbored potentially actionable variants in their tumors were classified into different evidence levels of predictive biomarkers. The proportions of OncoKB actionability with and without inclusion of TMB-H as a predictive biomarker to immunotherapy^16^ were 64% of patients (*n* = 6498 harbored at least one genomic variants with a variable highest level of clinical evidence, Level 1, 32%; Level 2, 1%; Level 3A, 1%; Level 3B, 13%; Level 4, 16%, As shown in Fig. 4a and Supplementary Fig. 13a) and 58% (*n* = 5899, Level 1, 17.9%; Level 2, 1.5%; Level 3A, 1.5%; Level 3B, 17.8%; Level 4, 19.2%), respectively. By removing samples with TMB-H, the number of Level 1 was reduced to 18%. To further investigate whether the remaining 3, 696 patients without OncoKB Level 1–4 variants in the OM cohort had an actionable biomarker, we analyzed PD-L1 expression. We found that 4% of these patients exhibited at least PD-L1 positive (Supplementary Fig. 13b), suggesting those patients could be candidates for treatment with immune checkpoint inhibitors even if their tumors did not meet the criteria for Level 1–4. A higher ratio of Level 1 was observed mainly in NSCLC, BRCA, SCLC, and UC, compared to that in other cancer types (Fig. 4b). Level 1 was predominantly represented by TMB-H and *EGFR* mutations in NSCLC, including *EGFR* L858R (20%; the proportion of samples in the tumor type with the variant), exon 19 deletion (19%) and G719 (3%) mutations. Others included *ALK* (7%) fusions in NSCLC, *PIK3CA* mutations (31%), and *ERBB2* amplification (24%) in BRCA and MSI-H in CRC (8%) (Fig. 4c). In terms of population-level mutation of actionable variants, *KRAS*, *EGFR* and *PIK3CA* SNVs/InDels, *ERBB2* amplification, and *ALK* fusions were most common, which was consistent with reports in the MSK cohort (Supplementary Fig. 13a). Interestingly, in NSCLC, TMB-H was negatively associated with fusion-positive (3 vs. 13% fusion frequency in TMB-H cohort and TMB-L cohort, respectively, *P* = 1.31E−11), mostly from *ALK* gene. In contrast, MSI-H showed a positive association with fusion-positive (6% vs. 1% fusion frequency in MSI-H cohort and MSS cohort, respectively, *P* = 0.04), mostly from *NTRK* gene, which hinted that clinical benefit of patients from the combination of fusion-based targeted therapy and immunotherapy is different in different types of cancers and the finding requires more studies to confirm in the future. All these findings suggested the relevance of treatment to the mutational landscape of Chinese tumor patients.

In conclusion, we report herein the somatic mutation landscape of over 10,000 solid tumors in Chinese patients. To our knowledge, this is the largest and most comprehensive mutational landscape analysis of solid tumors in an Asian population. This report provides a highly reliable dataset and resources for cancer medicine. More importantly, this population-level comparative analysis has comprehensively revealed similarities and differences between somatic alterations and actionable variants between Chinese and other ethnic populations with solid tumors and has an important implication for the selection of patients for clinical trials with molecularly targeted therapies.

## Methods

### Samples and patients

A total of 11,553 patients across 25 tumor types were submitted for NGS-based cancer assay (CSYS) in a Clinical Laboratory Improvement Amendments (CLIA)-certified and College of American Pathologists (CAP)-accredited laboratory (OrigiMed). Tumor types were annotated according to an institutional classification system, OncoTree (http://www.cbioportal.org/oncotree/).

Shanghai Ethics Committee For Clinical Research approved this study (“origimed-004”) on June 7, 2021 (Approval Number: SECCR2021-17-01). Patients were reimbursed for their participation. Besides, all the enrolled patients have signed an informed consent form to permit the use of biological samples and test results for the research. The representative cases can be found in the additional files and all raw copies have been deposited at the warrant website.

Unique tumor samples and matched normal blood samples of each patient were collected by standardized protocols. All tumor samples were formalin-fixed and paraffin-embedded (FFPE). Hematoxylin and eosin (H&E)-staining sections of tumor samples were reviewed by senior pathologists for the estimation of tumor cellularity. For each tumor sample, 15 to 25 eligible unstained sections were collected for DNA extraction. According to multiple quality control metrics, 825 (7%) samples with insufficient tumor content (<10%), 321 (3%) samples with inadequate extracted DNA yield (<50 ng), and 213 (2%) samples with a sequencing technical failure (unique mean coverage lower than 300×, biased coverage distribution or sample contamination) were excluded. In total, 10,194 (88%) samples were successfully included in the final analysis (Supplementary Fig. 1).

### Sequencing workflow

The laboratory and bioinformatics protocols of the CSYS panel had been described and validated in previous study^8^ (Supplementary Fig. 14). DNA extracted from tumor tissues and matched normal peripheral blood was fragmented to ~250 bp and subjected to library construction using KAPA HyperPrep Kits (KAPA Biosystems), followed by hybridization capture using custom xGen Lockdown Probes and Reagents (Integrated DNA Technologies). As the main component, the custom hybridization capture panel targets ~2.6 Mb of the human genome containing all coding exons of 450 genes (Supplementary Data 12), as well as the promoter of *TERT* and select introns of 39 genes frequently rearranged in cancer. Post-capture libraries were mixed, denatured and diluted to 1.5–1.8 pM (NextSeq 500) or 200–230 pM (NovaSeq 6000) and subsequently sequenced on NextSeq 500 or NovaSeq 6000 sequencers (Illumina). Paired-end sequencing was done following the manufacturer’s protocols. Tumor samples were sequenced to a median unique coverage of 1202× (Supplementary Fig. 3) and matched normal blood samples were sequenced to a mean unique coverage 300×. Data quality was inspected and controlled, followed by a suite of customized bioinformatics pipelines for variant calls. SNVs, InDels, and CNVs were identified using MuTect, Pindel, and EXCAVATOR, respectively. Gene rearrangements were detected using an algorithm developed in-house. At least 5 unique supporting reads were necessary for a SNVs/InDels. All variants were manually reviewed in the Integrative Genomics Viewer (IGV) and a custom visual software to avoid false positives. Test results, including somatic variants and inherited pathogenic variants, were returned to patients and their physicians based on their needs.

### Microsatellite instability (MSI) and tumor mutational burden (TMB)

MSI status and TMB of tumor samples are according to bioinformatics approaches developed in house^8^. Microsatellite instability-high (MSI-H) is defined as more than 15% of selected microsatellite loci showing unstable in tumors compared to matched peripheral blood. The TMB score of each tumor sample is calculated by counting the number of somatic SNVs and InDels per megabase (Mb) in the targeted coding region of the genome. Noncoding mutations, hotspot mutations and known germline polymorphisms in the U.S. National Center for Biotechnology Information’s Single Nucleotide Polymorphism Database (dbSNP) are not counted. In this study, 10 was adopted as the threshold value for differentiating TMB high (TMB-H) from TMB low (TMB-L).

### Overall comparative analysis pipeline

The available full data (mutation results and clinical information) of the MSK-IMPACT and TCGA (PanCancer Atlas and ovarian cancer, Nature 2011) studies were downloaded from cBioPortal (https://www.cbioportal.org/). The corresponding tumor types with >60 patients in each cohort were comparable. Somatic variants of OM and MSK datasets were comparatively analyzed, including somatic SNVs, InDels, deletions of tumor suppressor genes, amplifications of oncogenes, and functional fusions/rearrangements. Considering the differences in detecting methods between OM and TCGA studies, only a comparative analysis of somatic SNVs and InDels of the TCGA dataset was performed. These variants were in coding regions, exon–intron flanks and 5′ flanks (*TERT* gene) of 266 comparable cancer-related genes. All variants were divided into several subtypes, including SNVs/InDels, truncation, amplification, deletion, and fusion/rearrangement. Chi-squared test (*χ*²) and Fisher’s exact test were performed to the comparison of the frequencies of gene variants between two cohorts, and then *P* values were corrected with Benjamini–Hochberg (BH) method. Genes whose cohort-level altered frequency difference with statistic false discovery rate (FDR < 0.05) were reported as significant. We eliminated the influence of confounder factors by performing the comparative analysis (PSM) before comparing frequency. Be restricted to the available clinical information, only gender, smoking status [for LUAD, LUSC, and HNC only], sampling method, and primary/metastasis/recurrent tumor specimen were considered between aOM and MSK cohorts, and only gender, age, and primary/metastasis/recurrent tumor specimen between aOM and aTCGA cohorts. We tried to balance as many available confounder factors (primary/metastasis/recurrent tumor specimens, sampling method, gender, smoke, age, stage, grade, subtype of the tumor, depth of sequencing coverage, tumor purity, and patient ancestry) as possible. Given the limited availability of factors, this study will inevitably have some biases.

### Programmed death-ligand 1 (PD-L1) immunohistochemistry staining assay

We performed immunohistochemistry (IHC) staining of FFPE tissue sections for PD-L1 protein using an anti-PD-L1 antibody (clone 28-8; Cat#ab205921; Abcam; 1:300). Briefly, slides were incubated at 60 °C, deparaffinized in xylenes, and rehydrated with graded ethanol. Antigen retrieval was performed using the Universal HIER antigen retrieval reagent (Cat#ab208572; Abcam; 1:10) in a steamer. Non-specific binding was blocked with the Dako EnVision FLEX Peroxidase-Blocking Reagent. All other staining was performed primarily with Dako series reagents (Cat#K8002; Dako; Undiluted). All slides were counterstained with hematoxylin. Specimens were scored as positive by the pathologist using the  Tumor Proportion Score (TPS), which is the percentage of viable tumor cells with partial or complete membrane staining at any intensity. PD-L1 positivity in the study was defined as TPS ≥ 1%, and the specimens with 1–50% TPS and ≥50% TPS were respectively scored as weakly and strongly positive, respectively.

### Clinical utility evaluation

We used previously reported criteria in the MSK study to assess the clinical actionability of variants. OncoKB (August 31, 2021, http://oncokb.org/) knowledge base was used to annotate and classify variants into different levels: Food and Drug Administration (FDA)-recognized biomarkers (Level 1), variants that predict response to standard-of-care therapies (Level 2), variants that predict response to investigational agents in clinical trials (Level 3), or variants that predict to investigational agents in preliminary, preclinical studies (Level 4). These levels were also subdivided according to evidence within or between tumor types: Level A (1, 2A, 3A, 4) for the same tumor type, and Level B (2B, 3B) for different tumor types. Although wild-type *KRAS* was defined as a level-related factor, we excluded wild-type *KRAS* in CRC in this study when establishing the subset of Level 1. A high level of MSI-H was considered as an independent predictive biomarker with evidence of Level 1, regardless of the tumor type. If a variant was involved in different levels, the highest level was chosen for further analysis according to the rank: Level 1 > 2 > 3A > 3B > 4. The final level of each patient was defined as the highest evidence level of all variants detected in the patient. Information about drugs was from the U.S. Food and Drug Administration (FDA) (http://www.fda.gov) and the National Medical Products Administration (NMPA) of China (http://www.nmpa.gov.cn).

### Statistical analysis

Chi-squared test (*χ*²), Fisher’s exact test, and Benjamini–Hochberg (BH) method were used in the comparison of the gene alteration frequency between two cohorts, and they were also used to evaluate the association between clinical characteristics and significantly altered genes mutations. Corrections were also performed using the BH method. A Wilcoxon test could be done within each tumor type to compare the tumor mutational burden (TMB) between the MSK and the OM. The significant differences in this study were based on *P* values or FDR < 0.05.

### Reporting summary

Further information on research design is available in the Nature Research Reporting Summary linked to this article.

## Supplementary information

Supplementary Information

Reporting Summary

Supplementary Dataset 1

Supplementary Dataset 2

Supplementary Dataset 3

Supplementary Dataset 4

Supplementary Da5taset 5

Supplementary Dataset 6

Supplementary Dataset 7

Supplementary Dataset 8

Supplementary Dataset 9

Supplementary Dataset 10

Supplementary Dataset 11

Supplementary Dataset 12

## Data Availability

Public datasets used in this study include the MSK-IMPACT [https://www.cbioportal.org/study/summary?id=msk_impact_2017] and TCGA (PanCancer Atlas and ovarian cancer) [https://www.cbioportal.org/study/summary?id=ov_tcga_pub] studies were downloaded from cBioPortal. Variants were analyzed using the publicly available OncoKB knowledge database [http://oncokb.org]. Drug information was accessed from the U.S. Food and Drug Administration (FDA) [http://www.fda.gov] and the National Medical Products Administration (NMPA) of China [http://www.nmpa.gov.cn]. Multidimensional genomic and clinical data summaries in this study are accessible on the cBioPortal website [https://www.cbioportal.org/study/summary?id=pan_origimed_2020]. The raw data from patients are not publicly available due to restrictions on participant privacy and consent. Raw sequencing data may be obtained from the corresponding authors upon request. A transfer agreement and ethical review are required, which may take up to 3 months for the processing. Access will be granted for academic use only. Source data are provided in this paper.

## Source data

Source Data

## Code availability

All codes used in this manuscript are publicly available [http://ftp.origimed.com/gravityproject].
