# Supplementary material for: Landscape of somatic alterations in large-scale solid tumors from an Asian population
Source: Nat Commun. 2022 Jul 23;13:4264. doi: 10.1038/s41467-022-31780-9 (PMC9308789; doi:10.1038/s41467-022-31780-9)
Supplement: Supplementary file 1 — Supplementary Information [file 41467_2022_31780_MOESM1_ESM.pdf]

Supplementary Figures

Supplementary Figure 1

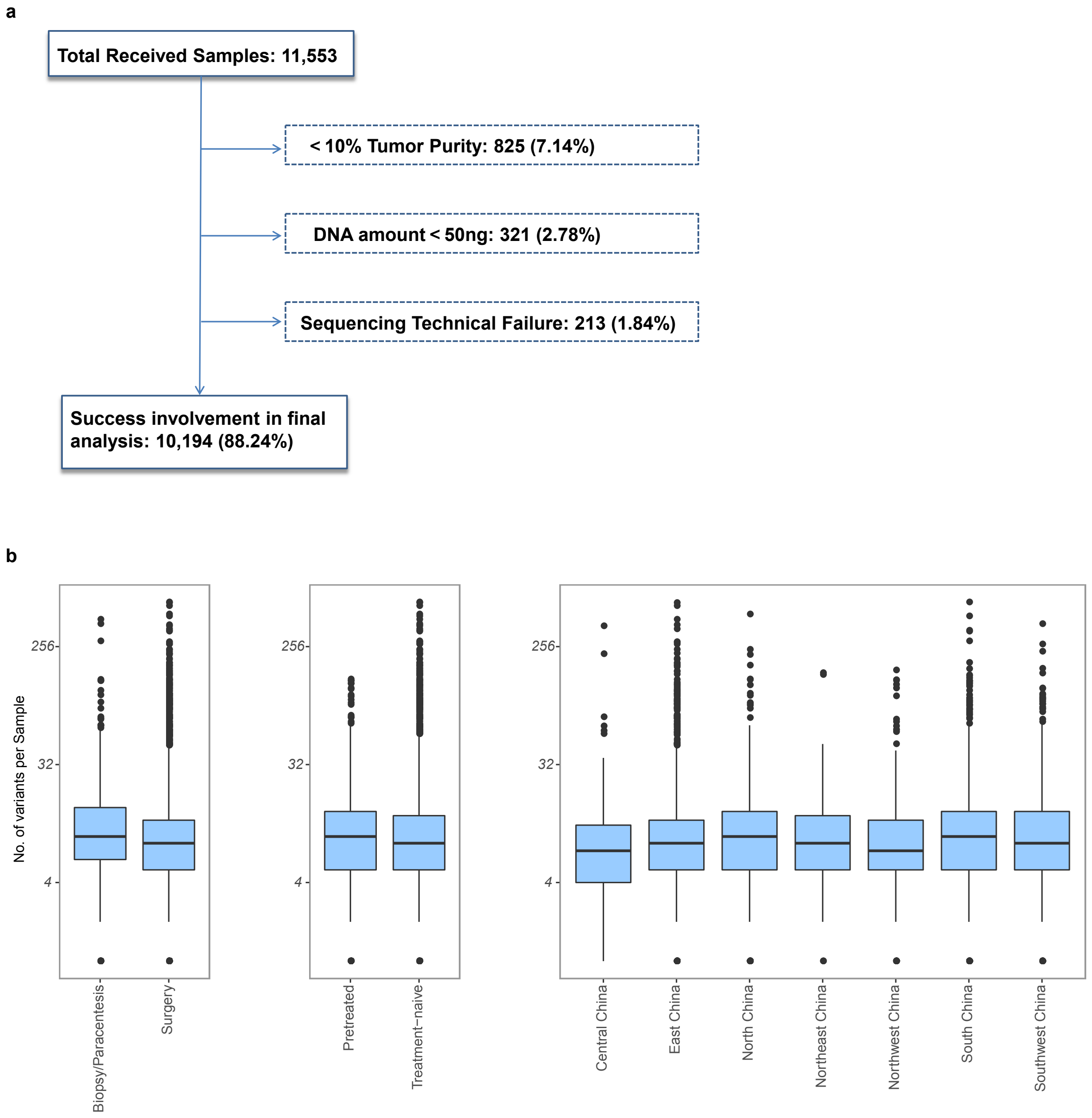

Supplementary Fig.1: (a) Selection processing of OM samples and success rates for analysis. A total of 11,553 tumor samples were submitted for NGS based cancer assay in the OrigiMed lab. A total of 1,359 samples were excluded in several quality control steps. Finally, 10,194 cases were successfully involved in final analysis with a success rate of 88%. (b) Variations per sample for different sampling methods, treatments and geographic origins. The box plots in a, c, and d show the minima, first quartile, median, third quartile, and the maxima. Distribution of Variations per sample in Biopsy/Paracentesis group (n=2418) and Srugery group (n=7606), Pretreated group (n=1608) and Treatment-naive group (n=7579), and Central_China group (n=492), East_China group (n=4144), North_China group (n=940), Northeast_China group (n=228), Northwest_China group (n=327), South_China group (n=2945), and Southwest_China group (n=948), respectively.

Supplementary Figure 2

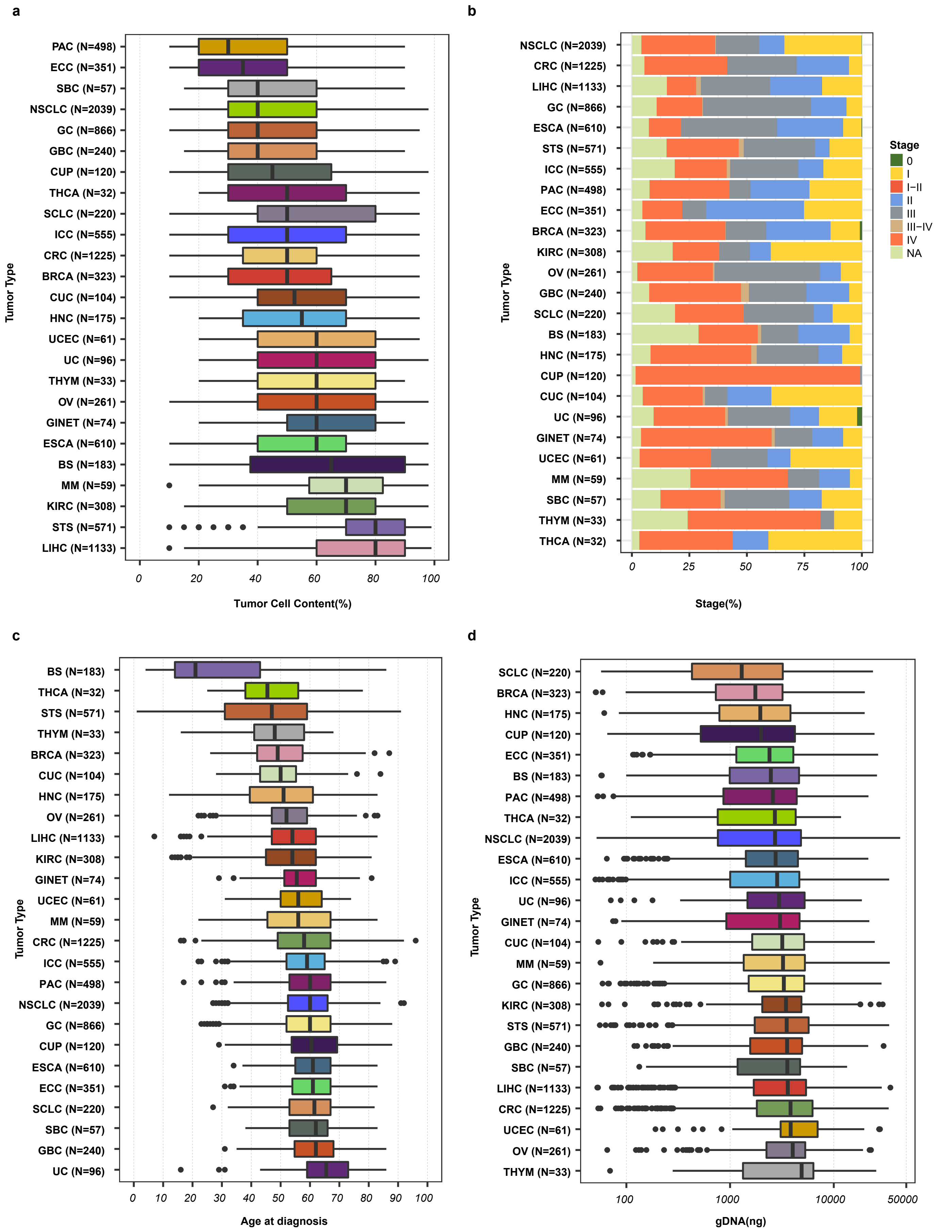

Supplementary Fig. 2: The tumor type-specific distribution of (a) tumor cell purity, (b) stage of disease, (c) age at diagnosis, and (d) genomic DNA (gDNA) content extracted from samples. The box plots in a, c, d show the minima, first quartile, median, third quartile, and the maxima.

Supplementary Figure 3

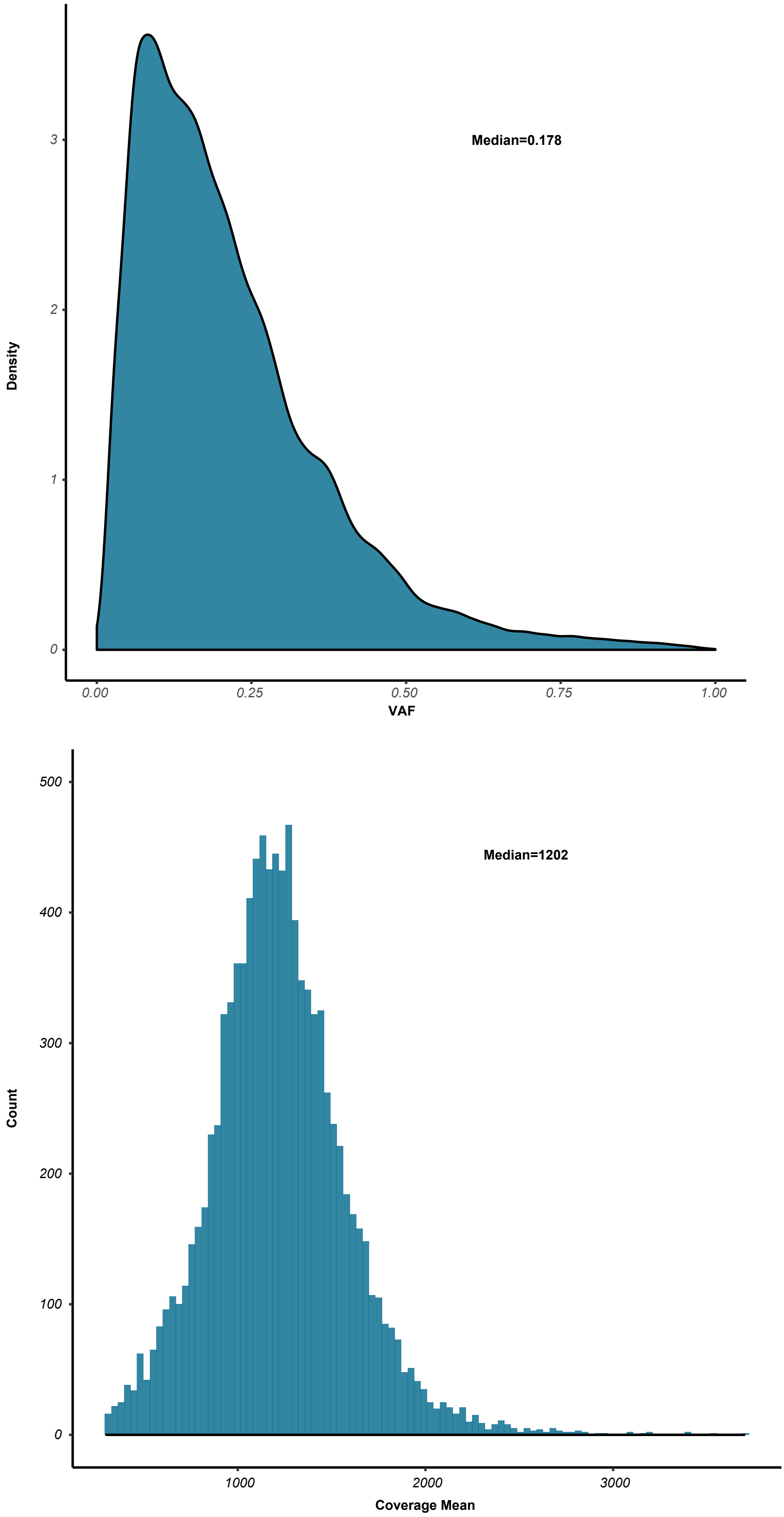

Supplementary Fig. 3: Distribution of variant allele frequency (VAF) and mean unique coverage. Distribution of VAF (≥ 2%) for mutations detected and reported in the OM dataset is shown above. Distribution of mean unique sequence coverage of 10,194 samples successfully sequenced is shown below.

Supplementary Figure 4

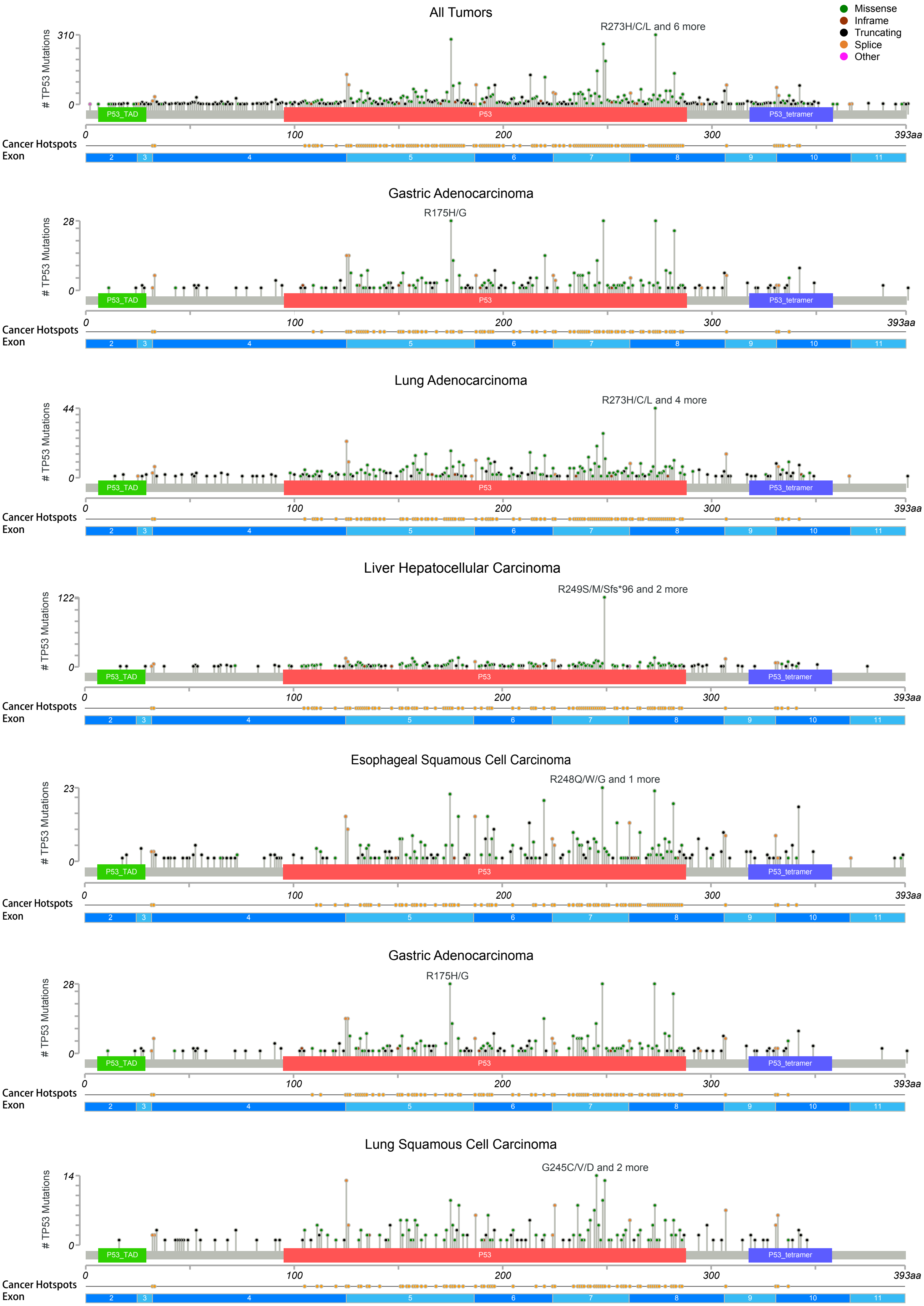

Supplementary Fig. 4: Location of *TP53* codon mutation in various tumor types.

Supplementary Figure 5

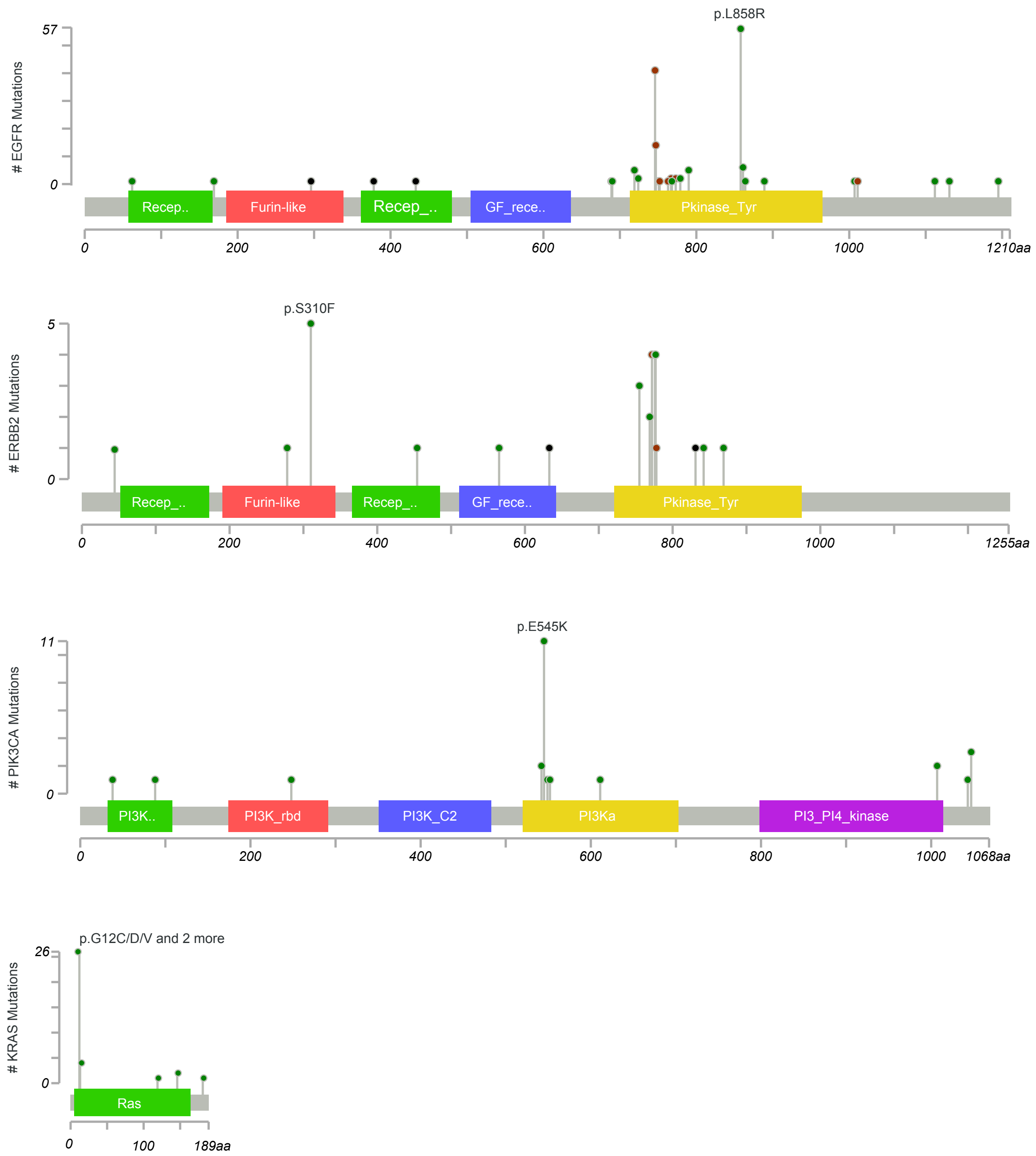

Supplementary Fig. 5: Location of *EGFR*, *ERBB2*, *PIK3CA* and *KRAS* gene mutations which were concomitant with amplification of corresponding genes.

Supplementary Figure 6

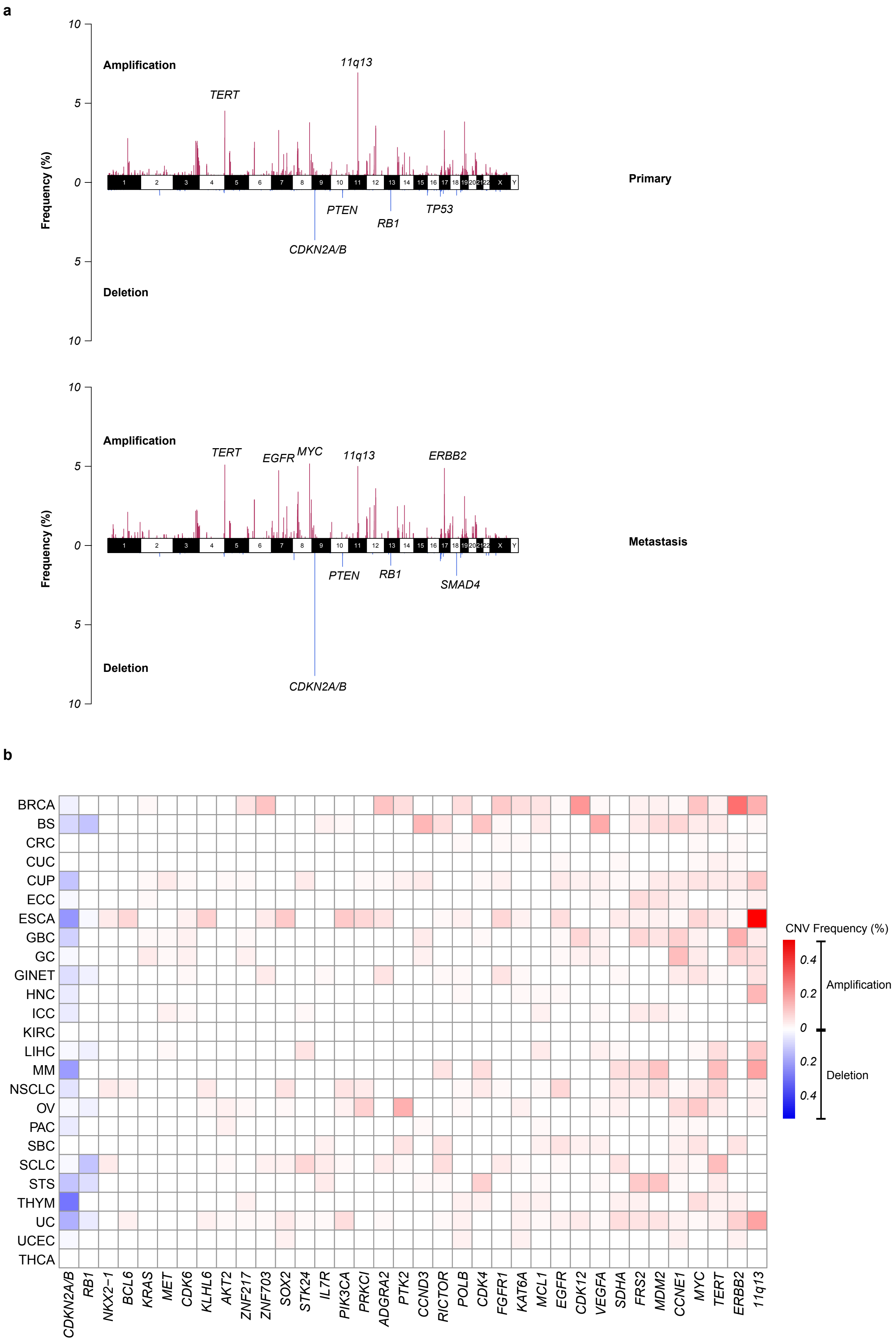

Supplementary Fig. 6: Distribution of CNVs in primary/ metastatic tumors and across tumor types. (a) Pan-cancer chromosome distribution of CNVs in primary and metastatic tumors. (b) The tumor type-specific distribution of recurrent CNVs. Red represents gene amplification and blue represents deletion. Frequencies are displayed in color gradient.

Supplementary Figure 7

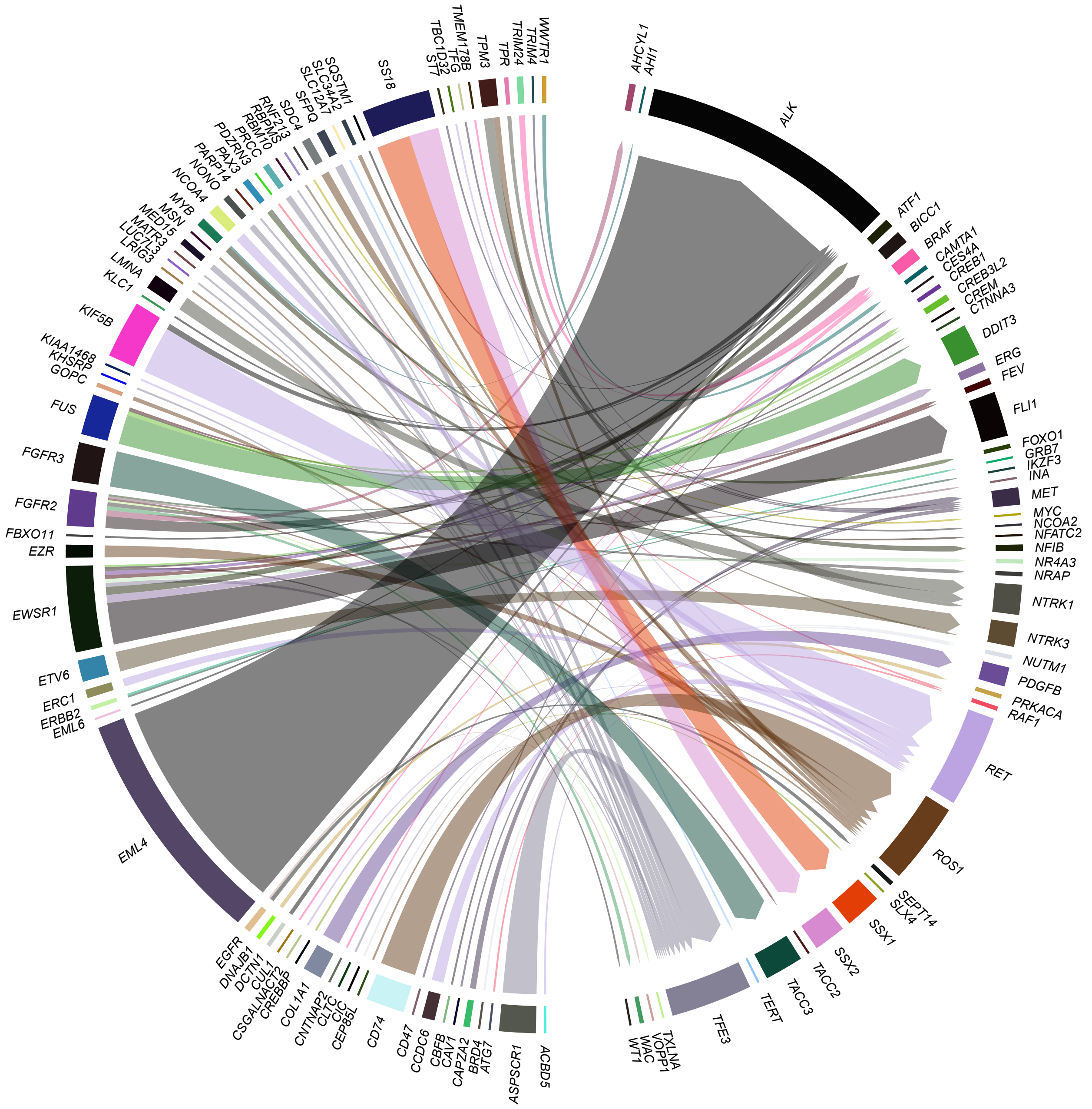

Supplementary Fig. 7: Spectrum of known fusions identified in OM cohort. Known recurrent gene fusion driver-partner relationships across tumor types are profiled. A total of 94 known relationships reported previously were detected spanning 115 genes. The thickness of the line between two genes implies the relative count.

Supplementary Figure 8

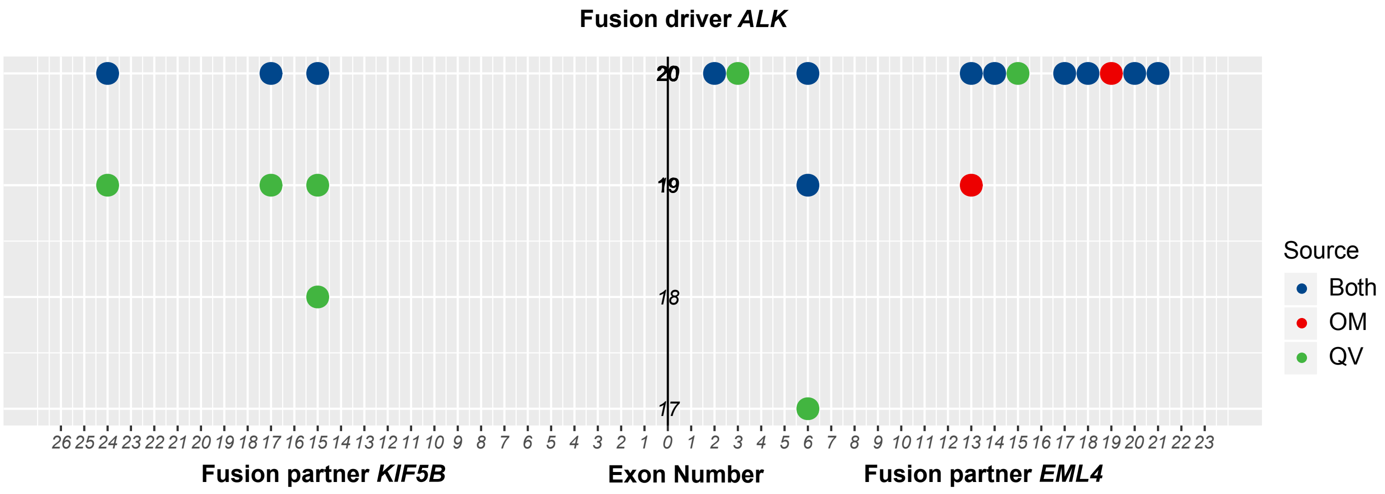

Supplementary Fig. 8: Fusion subtypes between *ALK* and *KIF5B* or *EML4*. The middle line represents the fusing exons of *ALK* gene; fusing exons of *KIF5B* and *EML4* are displayed at the left and right side, respectively. The exact fusion subtype relationships can be read according to the corresponding exons from *ALK* and *KIF5B* or *EML4*. Blue dots represent known fusions detected in both the Quiver dataset (http://quiver.archerdx.com/) and the OM dataset; red dots represent fusions only detected in the OM dataset; green dots represent fusions only found in the Quiver dataset.

Supplementary Figure 9

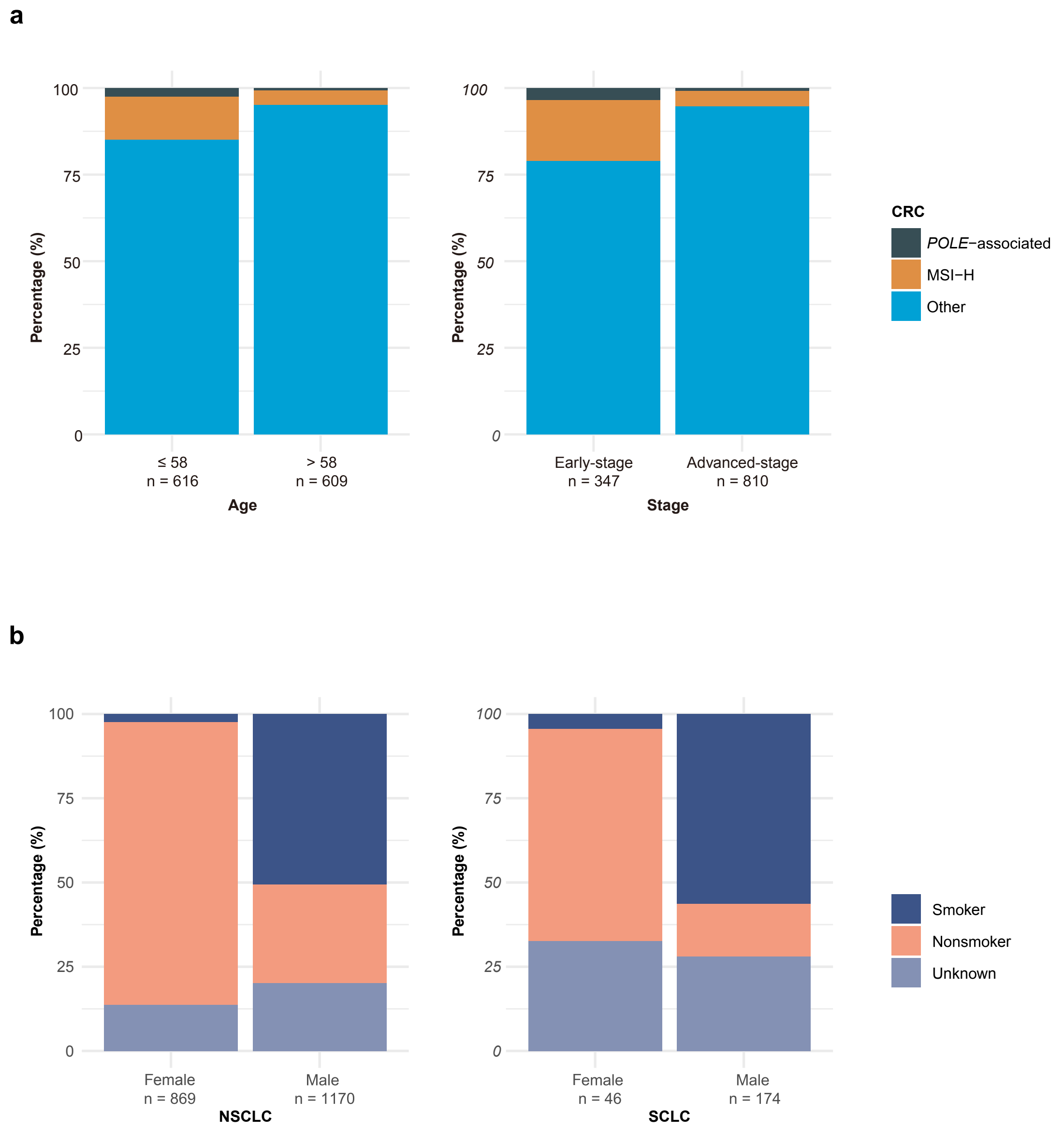

Supplementary Fig. 9: (a) Distribution of hypermutated subtypes of CRC and (b) correlation between gender and smoke feature in lung cancers. The median initial diagnosis age (58) of all CRC patients is used to separate into younger and older patients. *POLE*-associated CRC is defined as microsatellite stable (MSS) tumors with high mutation burden and at least one inactive POLE mutation. The "smoker" includes current smokers and former smokers, while "nonsmoker" means never smoker.

Supplementary Figure 10

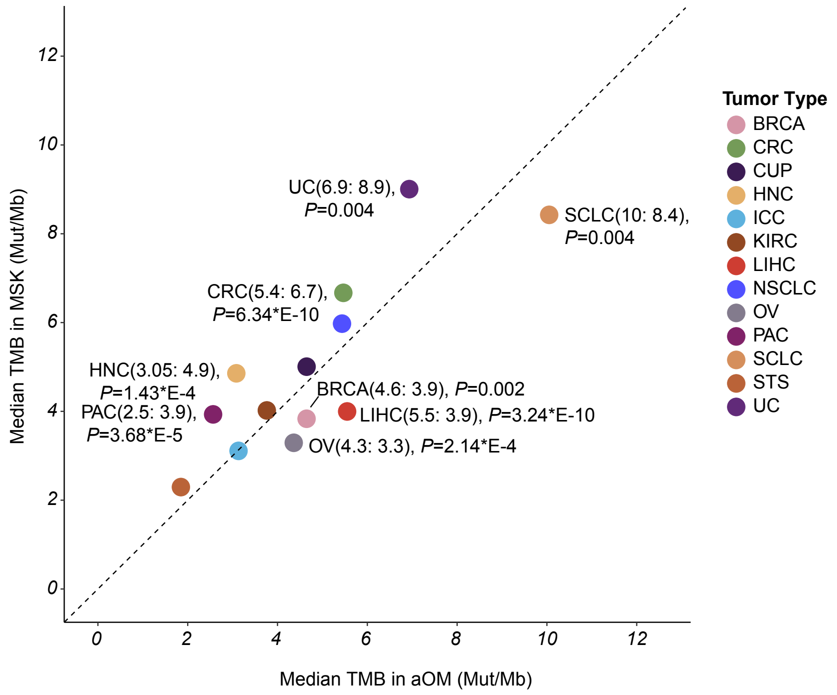

Supplementary Fig. 10: Comparative analysis of median TMB across various tumor types between the aOM cohort and MSK cohort. The median value of TMB of each tumor type (aOM: MSK) is shown. TMB values that were statistically significantly (by two-sided Wilcoxon-test, P < 0.01) different between the aOM cohort (x-axis) and the MSK cohort (y-axis) are labeled.

Supplementary Figure 11

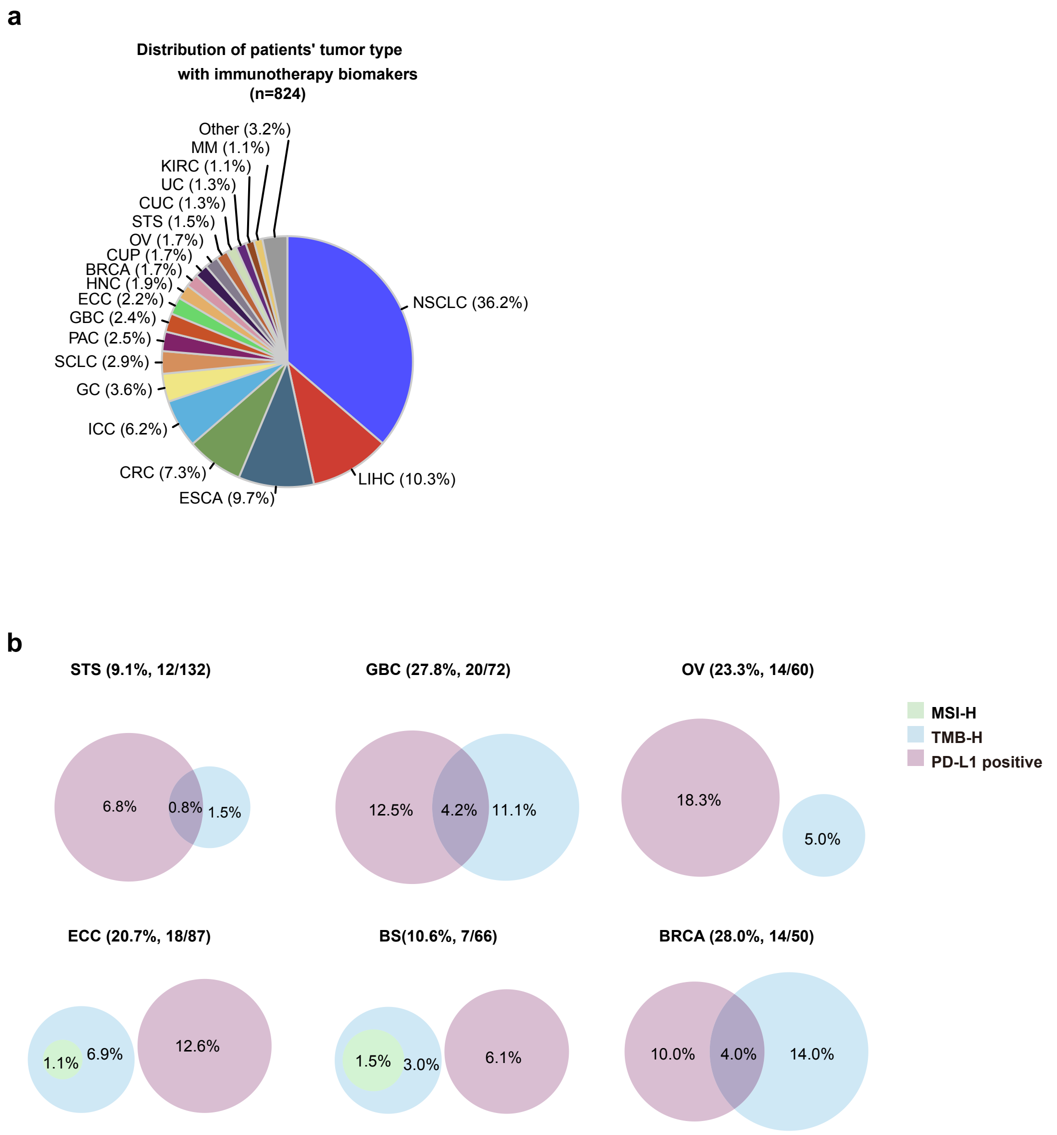

Supplementary Fig. 11: (a) Tumor type-specific distribution of 891 samples which were at least one of the following: MSI-H, TMB-H, PD-L1 positive, and (b) the association between PD-L1 positive and MSI-H and TMB-H in ECC, GBC, BS, OV, SCLC and BRCA.

Supplementary Figure 12

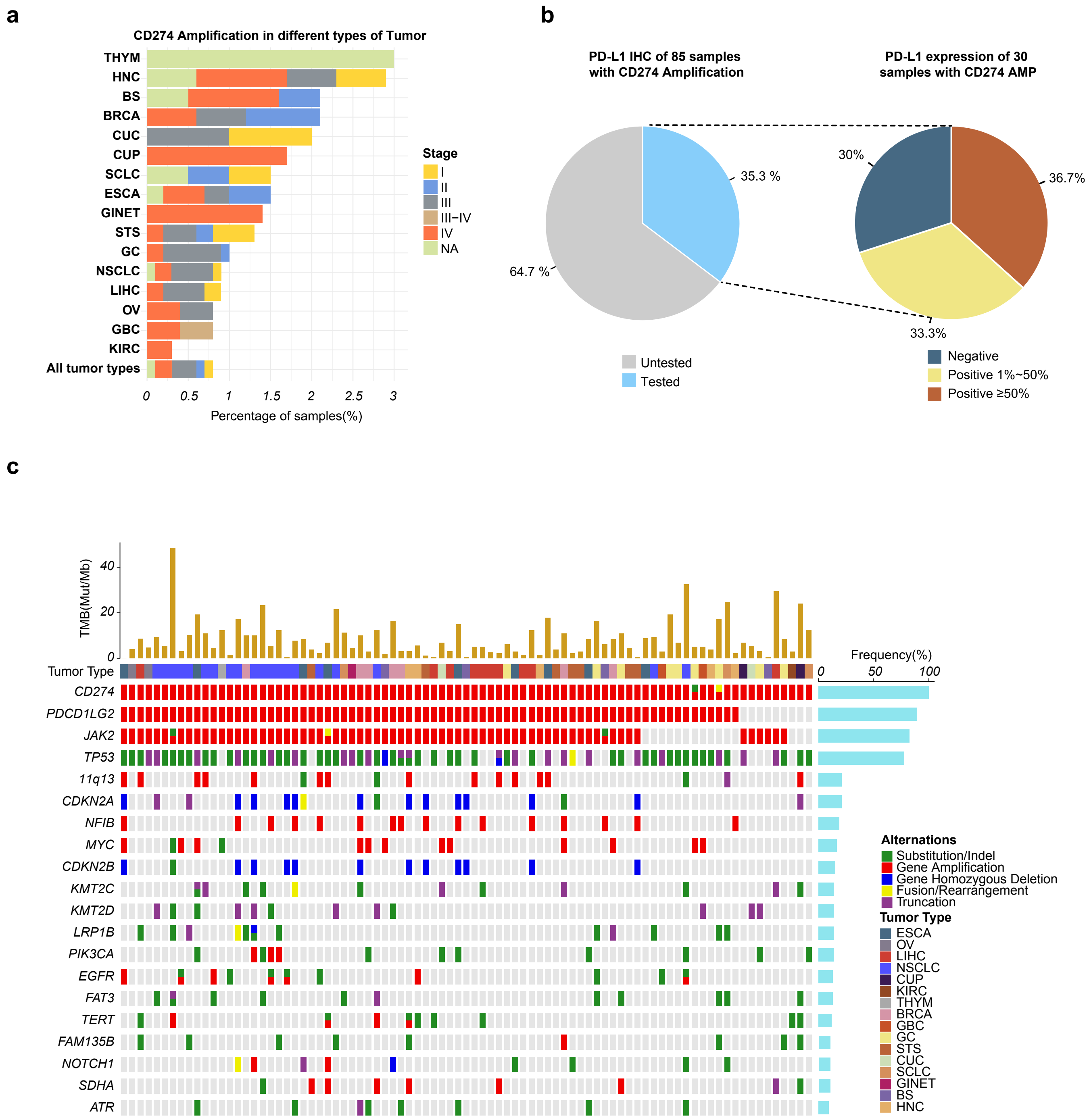

Supplementary Fig. 12: Significance of *CD274* amplification in Chinese patients with solid tumors. (a) The tumor type-specific distribution of tumors with *CD274* amplification is presented as the total or stage-specific percent of patients (%) across tumor types; stages are shown in different colors. (b) Expression of PD-L1 protein in tumors with *CD274* amplification. Thirty of 85 tumors with *CD274* amplification were tested for PD-L1 expression by IHC staining assay with Abcam 28-8 antibody. PD-L1 strong positive is defined as TPS > 50%. Among 30 tumors with tested PD-L1 protein, 70% showed positive expression. (c) The variation spectrum of 85 tumors with *CD274* amplification was profiled. The top 20 mutated genes are displayed in descending order. The upper panel represents the TMB value and the right panel indicates the frequencies of mutated genes.

Supplementary Figure 13

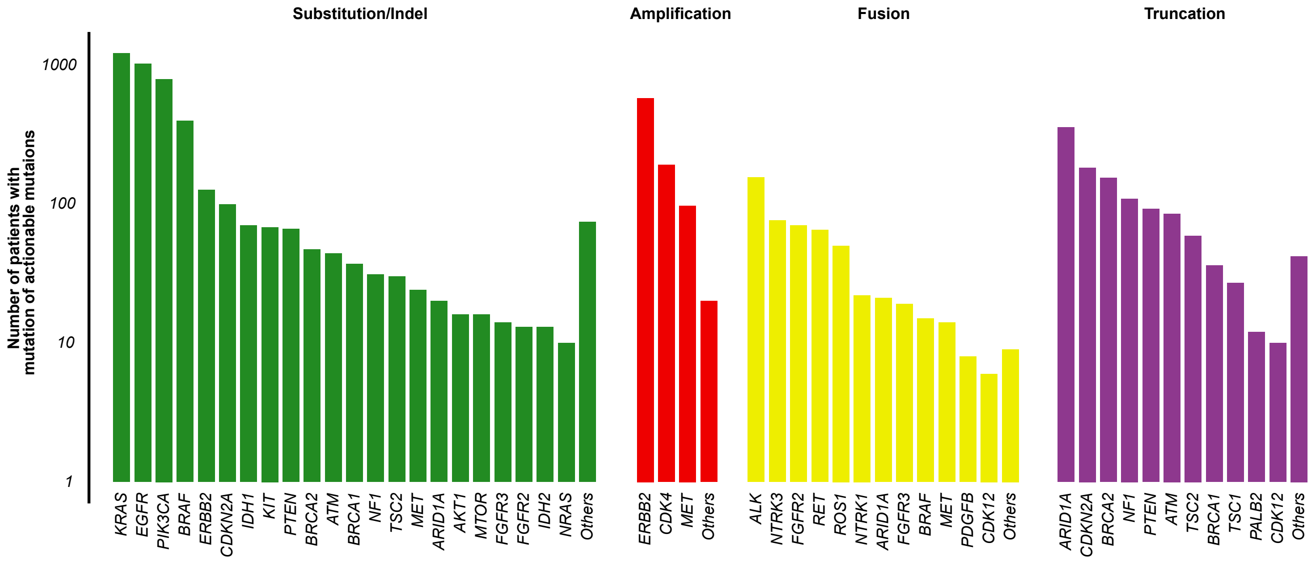

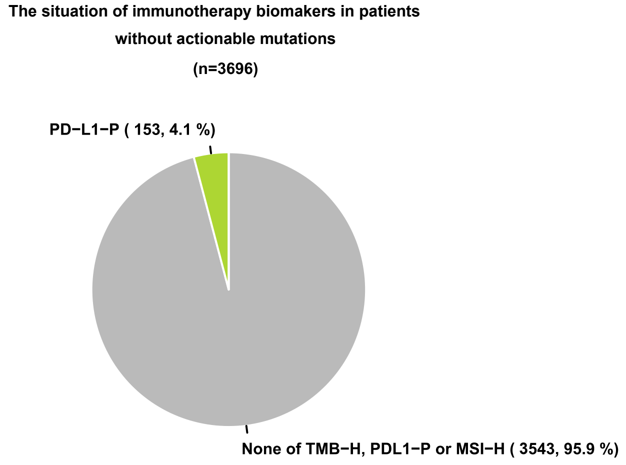

Supplementary Fig. 13: (a) Number of patients with clinical actionable variants according to four variant subtypes including substitution/indel, amplification, fusion/rearrangement, and truncation. (b) The distribution of TMB-H and PD-L1 positive in patients without clinical actionable variants.

Supplementary Figure 14

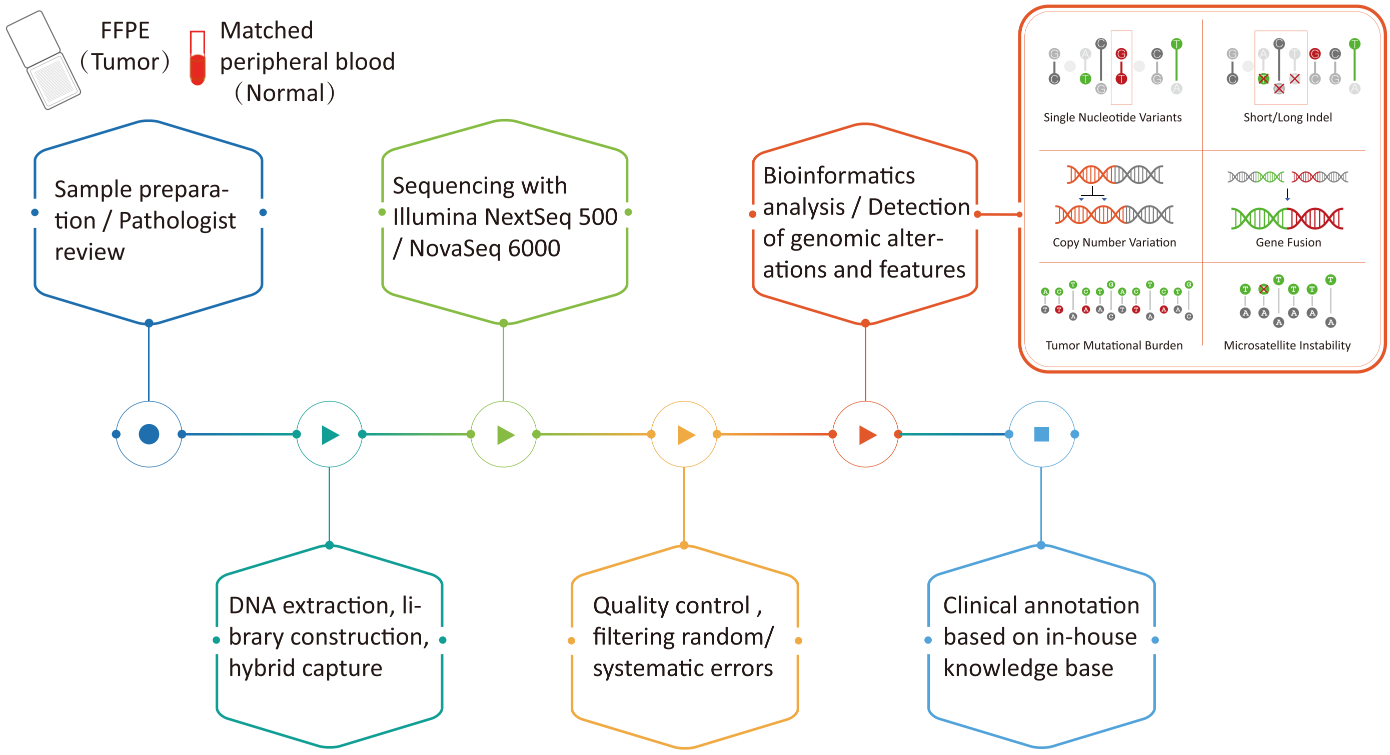

Supplementary Fig. 14: Overview of the NGS based cancer assay (CSYS) workflow.
