# Supplementary material for: Landscape of somatic alterations in large-scale solid tumors from an Asian population
Source: Nat Commun. 2022 Jul 23;13:4264. doi: 10.1038/s41467-022-31780-9 (PMC9308789; doi:10.1038/s41467-022-31780-9)
Supplement: Supplementary file 2 — Reporting Summary [file 41467_2022_31780_MOESM2_ESM.pdf]

nature portfolio

Corresponding author(s): Kai Wang

Last updated by author(s): Jun 9, 2022

Reporting Summary

Nature Portfolio wishes to improve the reproducibility of the work that we publish. This form provides structure for consistency and transparency in reporting. For further information on Nature Portfolio policies, see our Editorial Policies and the Editorial Policy Checklist.

Statistics

For all statistical analyses, confirm that the following items are present in the figure legend, table legend, main text, or Methods section.

n/a	Confirmed	
☐	☒	The exact sample size (*n*) for each experimental group/condition, given as a discrete number and unit of measurement
☐	☒	A statement on whether measurements were taken from distinct samples or whether the same sample was measured repeatedly
☐	☒	The statistical test(s) used AND whether they are one- or two-sided<br>*Only common tests should be described solely by name; describe more complex techniques in the Methods section.*
☒	☐	A description of all covariates tested
☐	☒	A description of any assumptions or corrections, such as tests of normality and adjustment for multiple comparisons
☐	☒	A full description of the statistical parameters including central tendency (e.g. means) or other basic estimates (e.g. regression coefficient) AND variation (e.g. standard deviation) or associated estimates of uncertainty (e.g. confidence intervals)
☐	☒	For null hypothesis testing, the test statistic (e.g. *F*, *t*, *r*) with confidence intervals, effect sizes, degrees of freedom and *P* value noted<br>*Give P values as exact values whenever suitable.*
☒	☐	For Bayesian analysis, information on the choice of priors and Markov chain Monte Carlo settings
☒	☐	For hierarchical and complex designs, identification of the appropriate level for tests and full reporting of outcomes
☒	☐	Estimates of effect sizes (e.g. Cohen's *d*, Pearson's *r*), indicating how they were calculated

Our web collection on statistics for biologists contains articles on many of the points above.

Software and code

Policy information about availability of computer code

Data collection	No software was used for data collection.
Data analysis	The next-generation sequencing (NGS)-based CSYS panel sequence data were mapped to the human genome (hg19) using BWA aligner v0.5.9. PCR duplicate read removal and sequence metric collection was done using Picard 1.47 and Samtools 0.1.12a. Local alignment optimization was performed using GATK 1.0.4705. All types of genomic alterations (including Substitution/Indel, Gene Amplification, Gene Homozygous Deletion, Truncation and Fusion/Rearrangement) and the calculations of TMB and MSI were called using a suite of bioinformatics pipelines described previously in our published paper (Oncologist 2019 doi: 10.1634/theoncologist.2019-0236). R version 3.5.1 including some functions such as circos (circlize 0.4.7), venn (eulerr v5.1.0) and OncoPrint (ComplexHeatmap v2.1.0) was used for statistical analyses or graphic presentation. PSM (Propensity Score Matching)(MatchIt v4.3.2) was performed to adjust confounders when comparing the OM cohort with the MSK/TCGA cohort.

For manuscripts utilizing custom algorithms or software that are central to the research but not yet described in published literature, software must be made available to editors and reviewers. We strongly encourage code deposition in a community repository (e.g. GitHub). See the Nature Portfolio guidelines for submitting code & software for further information.

Data

Policy information about availability of data

All manuscripts must include a data availability statement. This statement should provide the following information, where applicable:

- Accession codes, unique identifiers, or web links for publicly available datasets
- A description of any restrictions on data availability
- For clinical datasets or third party data, please ensure that the statement adheres to our policy

public datasets used in this study include the MSK-IMPACT and TCGA (PanCancer Atlas and ovarian cancer, Nature 2011) studies were downloaded from cBioPortal (https://www.cbioportal.org/), OncoKB (August 31, 2021, http://oncokb.org/) knowledge base,the U.S. Food and Drug Administration (FDA) (http://www.fda.gov) and National Medical Products Administration (NMPA) of China (http://www.nmpa.gov.cn). All results data in this study are accessible on cbioportal (https://www.cbioportal.org/study/summary?id=pan_origimed_2020) and codes in http://ftp.origimed.com/gravityproject. All these mentioned in the manuscript under the "Data Availability" and "Code Availability".

Field-specific reporting

Please select the one below that is the best fit for your research. If you are not sure, read the appropriate sections before making your selection.

☒ Life sciences ☐ Behavioural & social sciences ☐ Ecological, evolutionary & environmental sciences

For a reference copy of the document with all sections, see nature.com/documents/nr-reporting-summary-flat.pdf

Life sciences study design

All studies must disclose on these points even when the disclosure is negative.

Sample size	No sample size calculated in this study. Based on the distribution of common tumor types with higher morbidity and mortality in the Chinese population, over 10,000 patients were successfully enrolled and currently this is the largest sample size to study Asian population is enough to our robust conclusions.
Data exclusions	Samples with tumor purity less than 10%, DNA amount less than 50ng or sequencing failed were excluded from the analysis.
Replication	All the NGS data were from patients, and no technical replication was done for each sample.
Randomization	Samples were collected like pan-cancer real world study. This study didn't control the covariates.
Blinding	The investigators were blinded to group allocation during data collection.

Reporting for specific materials, systems and methods

We require information from authors about some types of materials, experimental systems and methods used in many studies. Here, indicate whether each material, system or method listed is relevant to your study. If you are not sure if a list item applies to your research, read the appropriate section before selecting a response.

Materials & experimental systems

n/a	Involved in the study	
☐	☒	Antibodies
☒	☐	Eukaryotic cell lines
☒	☐	Palaeontology and archaeology
☒	☐	Animals and other organisms
☐	☒	Human research participants
☒	☐	Clinical data
☒	☐	Dual use research of concern

Methods

n/a	Involved in the study	
☒	☐	ChIP-seq
☒	☐	Flow cytometry
☒	☐	MRI-based neuroimaging

Antibodies

Antibodies used	Anti-PD-L1 antibodies (clone 28-8; Cat#ab205921; Abcam)
Validation	Accurately quantify PD-L1 with high sensitivity is stated on abcam website (https://securedrtest.abcam.com/kits/accurately-quantify-pd-l1-in-90-minutes). More than 300 elevant citations are list on website (https://www.abcam.com/pd-l1-antibody-28-8-ab205921.html?productWallTab=ShowAll), and antibody profile is showed in database (https://www.abcam.com/hrp-pd-l1-antibody-28-8-ab209961.html, https://www.uniprot.org/uniprot/Q9NZQ7). Primary antibodie is validated and titrated with appropriate positive and negative controls in order to determine the optimal stain concentration for each test.

Human research participants

Policy information about studies involving human research participants

Population characteristics	We collected a total of 11,553 tumor specimens and matched peripheral blood specimens from 11,553 individuals encompassing 25 principal tumor types and >100 tumor subtypes. The median age was 56-year old (range: 1-96). Male was accounted for 60.4% of the participants, while the female accounted for 39.6%.
Recruitment	From Nov. 2016 to Aug. 2019, 10000 patients were enrolled from the company, which is cooperated with multi center hospitals. There was no restrictions for patients' inclusion and exclusion. All samples with sufficient tumor content or DNA yield will be used for further detection.
Ethics oversight	This study was approved by the Shanghai Ethics Committee for Clinical Research (SECCR; approval number: SECCR2021-17-01). All patients gave informed consent to participate in the study and gave permission for the use of samples.

Note that full information on the approval of the study protocol must also be provided in the manuscript.
